# Do the receptive fields in the primary visual cortex span a variability over the degree of elongation of the receptive fields?

**DOI:** 10.1007/s10827-025-00907-4

**Published:** 2025-06-20

**Authors:** Tony Lindeberg

**Affiliations:** Computational Brain Science Lab, Division of Computational Science and Technology, KTH Royal Institute of Technology, SE-100 44, Stockholm, Sweden

**Keywords:** Receptive field, Elongation, Orientation selectivity, Simple cell, Complex cell, Pinwheel

## Abstract

This paper presents the results of combining (i) theoretical analysis regarding connections between the orientation selectivity and the elongation of receptive fields for the affine Gaussian derivative model with (ii) biological measurements of orientation selectivity in the primary visual cortex to investigate if (iii) the receptive fields can be regarded as spanning a variability in the degree of elongation. From an in-depth theoretical analysis of idealized models for the receptive fields of simple and complex cells in the primary visual cortex, we established that the orientation selectivity becomes more narrow with increasing elongation of the receptive fields. Combined with previously established biological results, concerning broad vs. sharp orientation tuning of visual neurons in the primary visual cortex, as well as previous experimental results concerning distributions of the resultant of the orientation selectivity curves for simple and complex cells, we show that these results are consistent with the receptive fields spanning a variability over the degree of elongation of the receptive fields. We also show that our principled theoretical model for visual receptive fields leads to qualitatively similar types of deviations from a uniform histogram of the resultant descriptor of the orientation selectivity curves for simple cells, as can be observed in the results from biological experiments. To firmly investigate the validity of the underlying working hypothesis, we finally formulate a set of testable predictions for biological experiments, to characterize the predicted systematic variability in the elongation over the orientation maps in higher mammals, and its relations to the pinwheel structure.

**Supplementary Information:**

The online version contains supplementary material available at 10.1007/s10827-025-00907-4.

## Introduction

**Fig. 1:**
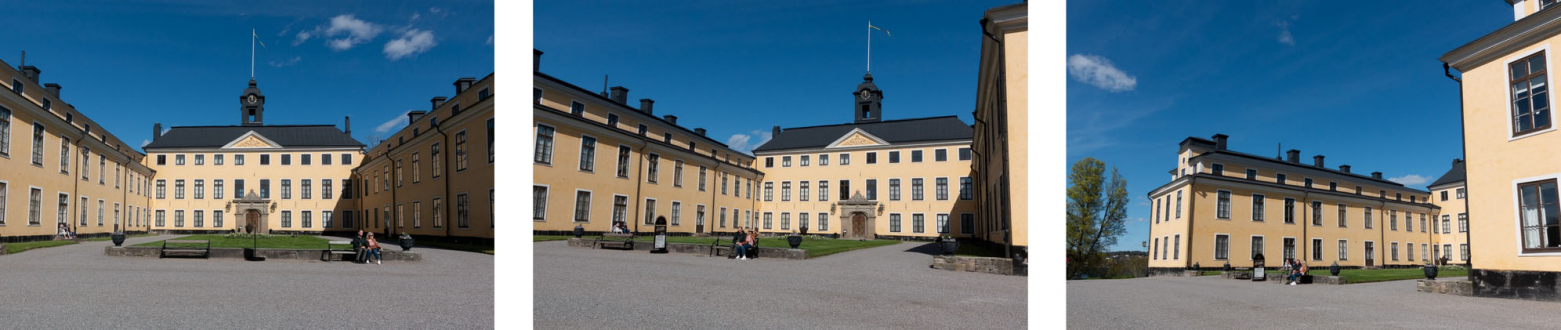
Variabilities in image structures generated by viewing the same surface patterns from different viewing directions. Observe how the resulting perspective transformations lead to strong foreshortening effects, in that the image structures in one direction in the 2-D image space are compressed more than the image structures in the orthogonal direction. Here, where the viewpoint of the observer is moved horizontally in the world, the foreshortening effect is mainly along the horizontal direction, although complemented also with small rotations for non-central image points, because of using a planar image plane as opposed to a spherical retina. To first order of approximation of the projective mappings between pairwise views, these resulting image deformations can be modelled in terms of local affine transformations

When observing objects and events in our natural environment, the image structures in the visual stimuli will be subject to substantial variabilities caused by the natural image transformations. Specifically, if observing a smooth local surface patch from different viewing directions and viewing distances, this variability can, to first order of approximation, be approximated by local affine transformations (the derivative of the projective mappings between the different views). Within the degrees of freedom of 2-D spatial affine transformations^1^ between two views of the same local surface patch, there is one degree of freedom that corresponds to non-uniform spatial scaling transformations with different amounts of scaling along two orthogonal directions. Geometrically, this degree of freedom corresponds to variabilities in the slant angle between the local surface normal and the viewing direction, as illustrated in Fig. 1 with multiple views of the same scene, obtained by moving the viewing point and the viewing direction in a horizontal plane.

**Fig. 2:**
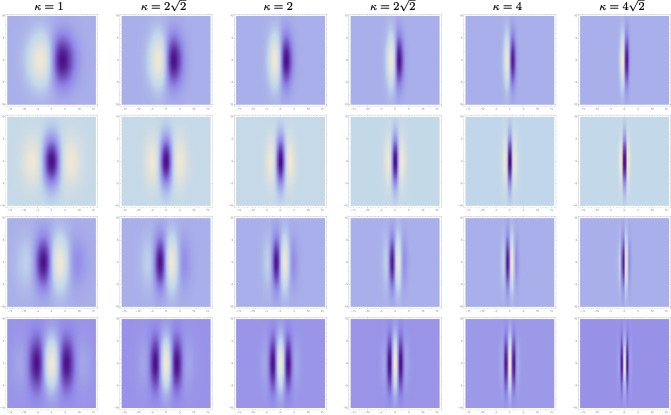
Variability in the elongation of affine Gaussian derivative receptive fields (for the image orientation $\varphi = 0$), with the scale parameter ratio $\kappa = \sigma _2/\sigma _1$ increasing from 1 to $4\sqrt{2}$ according to a logarithmic distribution, from left to right, with the vertical scale parameter kept constant $\sigma _2 = 4$ and with the horizontal scale parameter being the smaller $\sigma _1 \le \sigma _2$. (first row) First-order directional derivatives of affine Gaussian kernels according to Eq. (5) for $m = 1$. (second row) Second-order directional derivatives of affine Gaussian kernels according to Eq. (5) for $m = 2$. (third row) Second-order directional derivatives of affine Gaussian kernels according to Eq. (5) for $m = 3$. (fourth row) Second-order directional derivatives of affine Gaussian kernels according to Eq. (5) for $m = 4$. (Horizontal axes: image coordinate $x_1 \in [-16, 16]$. Vertical axes: image coordinate $x_2 \in [-16, 16]$)

If we consider a vision system that views 3-D scenes from multiple viewing directions, then the receptive field responses will be strongly influenced by geometric image transformations. A particular requirement on a vision system, as imposed by the requirement of affine covariance (to be described in more detail in Sections 3.1–3.2), is that the vision system should be able to match the outputs from the receptive fields between different views of the same scene. Then, under the foreshortening transformations that arise from varying the slant angles under variations in the viewing conditions of 3-D objects, the spatial shapes of the receptive fields would have to be adapted to the resulting non-uniform scaling transformations, to result in different eccentricities of the receptive fields, as illustrated in Fig. 2.

In Lindeberg (2021, 2023, 2025b, 2025a), we have outlined a general framework for how covariance properties with respect to geometric image transformations may constitute a fundamental constraint for the receptive fields in the primary visual cortex of higher mammals. The underlying aim is to enable the visual computations to be robust under the variabilities in the image structures generated by the natural image transformations. The fundaments of this theory are based on axiomatically determined receptive field shapes, derived from symmetry properties, that reflect structural properties of the environment, in combination with additional constraints to guarantee consistency between image representations over multiple spatial and temporal scales.

The population of receptive fields in the primary visual cortex ought to, according to this theory, obey covariance properties with respect to spatial affine transformations and Galilean transformations. Specifically, according to this theory, the receptive field shapes ought to be expanded over the degrees of freedom that correspond to the variabilities in image structures caused by these classes of geometric image transformations. Alternatively, the vision system ought to implement computational mechanisms that would to a sufficient good degree of approximation be computationally equivalent to such an expansion of receptive field shapes over the degrees of freedom of geometric image transformations. Notably, the expansion of the number of receptive fields, from about 1 M output channels from the lateral geniculate nucleus to the primary visual cortex (V1) to 190 M neurons in V1 with 37 M output channels (see DiCarlo et al., 2012 Figure 3), would indeed be consistent with an expansion of the receptive field shapes in V1 over parameters of the receptive fields.

While overall qualitative comparisons between predictions from this principled theory have been successfully made to neurophysiological recordings of receptive fields of simple cells by DeAngelis et al. (1995); DeAngelis and Anzai (2004); Conway and Livingstone (2006) and Johnson et al. (2008), publicly available data regarding full receptive field recordings are quite limited. Thus, further experimental evidence would be needed to firmly either reject or support the stated hypotheses about affine covariance and Galilean covariance.

In the lack of such neurophysiological data regarding full receptive field recordings, one could, however, aim to instead obtain indirect cues regarding a possible variability in the degree of elongation of the receptive fields, by making use of the recordings of the orientation selectivity of visual neurons in previous studies: Experimental results by Nauhaus et al. (2008) show a substantial variability regarding broad vs. sharp tuning of the receptive fields in the primary visual cortex. Goris et al. (2015) report comparably uniform distributions of the degree of orientation selectivity of simple and complex cells, in terms of histograms of the resultant of the orientation selectivity curves.

In a companion paper (Lindeberg, 2025), we have established a strong direct link between the orientation selectivity and the elongation of the receptive fields according to the idealized generalized Gaussian derivative model for visual receptive fields (as will be summarized in Section 3.3). If the generalized Gaussian derivative shape is indeed a good model for simple and complex cells in V1, then variations in their elongation would logically correspond to a variations in their orientation selectivity.

Thereby, these results together are fully consistent with the hypothesis that the visual receptive fields in the primary visual cortex should span^2^ a variability in their anisotropy, thus consistent with the hypothesis that the receptive fields should span at least one more degree of freedom in the affine group, beyond mere rotations (as can be established by the expansion of orientation selectivity properties around the centers of the pinwheel structures in the primary visual cortex of higher mammals).

To ultimately judge is this hypothesis would hold, we will finally use predictions from the presented theoretical analysis to formulate a set of explicit, testable hypotheses, that could be either verified or rejected in future neurophysiological experiments. Additionally, we will formulate a set of quantitive measurements to be made, to characterize a possible variability in the anisotropy or elongation of receptive fields in the primary visual cortex, with special emphasis on the relationships between a possibly predicted variability in receptive field elongation and the pinwheel structure in the primary visual cortex of higher mammals.

### Contributions and novelty

In summary, the purposes of this paper are twofold:

- In the current absence of firm biological measurements about a possible variability of the degree of elongation of receptive field shapes in the primary visual cortex, provide potential indirect support for such a hypothesis. This reasoning is based on a combination of previously established variability in the degree of the orientation selectivity of biological receptive fields with a model-based connection between the degree of elongation of the receptive fields and their orientation selectivity.
- To formulate a set of theoretically motivated and experimentally testable predictions and quantitative measurements, that could be used by experimentalists for ultimately judging whether the formulated hypothesis about a variability over the degree of elongation of the receptive fields would hold in the primary visual cortex of higher mammals. Such a connection could then provide potential further support for the more general hypothesis, that the receptive fields in the primary visual cortex of higher mammals may be covariant under the larger group of spatial affine transformations.

The main novel contributions of this paper are specifically:

- A qualitative explanation of the experimental results by Nauhaus et al. (2008) as the results of a systematic variability in the degree of elongation of the receptive fields.
- Biological implications of that result, in that the pinwheel structure of higher mammals should, beyond an expansion over image orientations, also comprise an expansion over the degree of elongation of the receptive fields, from the center of each pinwheel to its periphery.
- Theoretical connections to, as well as potential partial support for, the hypothesis that the receptive fields in the primary visual cortex may be covariant under spatial affine transformations.
- The quantitative explanation of the receptive field histograms obtained experimentally by Goris et al. (2015), as the results of combining the receptive fields of simple cells corresponding to different orders of spatial differentiation.
- Biological implications of that result, that the experimentally recorded receptive field histograms are better explained by receptive fields in terms of spatial derivatives up to order 4 than in terms of spatial derivatives up to order 2.
- The overall theoretical contributions in the paper, of explaining properties of the primary visual cortex, based on properties of a theoretically principled model for the receptive fields, using mathematical analysis and not numerical simulations of neural models as the main tool.

A further underlying motivation of this work is to lay out a conceptual foundation, by which theoreticians and experimentalists could join efforts to establish to what extent the distributions of the shapes of the biological receptive fields would be compatible with an explanation from the fundamental constraint, that the family of receptive fields should be able to handle variabilities in the image data caused by geometric image transformations.

## Related work

Beyond the works by Nauhaus et al. (2008) and by Goris et al. (2015), that the treatment in Section 4 will largely build upon, there is a large body of work on characterizing the orientation selectivity of neurons, by Watkins and Berkley (1974); Rose and Blakemore (1974); Schiller et al. (1976); Albright (1984); Ringach et al. (2002); Nauhaus et al. (2008); Scholl et al. (2013); Sadeh and Rotter (2014) and Sasaki et al. (2015), as well as concerning biological mechanisms for achieving orientation selectivity by Somers et al. (1995); Sompolinsky and Shapley (1997); Carandini and Ringach (1997); Lampl et al. (2001); Ferster and Miller (2000); Shapley et al. (2003); Seriès et al. (2004); Hansel and van Vreeswijk (2012); Moldakarimov et al. (2014); Cogno and Mato (2015); Priebe (2016); Pattadkal et al. (2018); Nguyen and Freeman (2019); Merkt et al. (2019); Wei et al. (2022) and Wang et al. (2024). The focus of this paper, however, is not on the neural mechanisms that lead to orientation selectivity, but on purely *functional properties* at the macroscopic level.

Mathematical models of biological receptive fields have been formulated in terms of Gaussian derivatives (Koenderink, 1984; Koenderink and van Doorn, 1987, 1992); Young and his co-workers 1987; Young et al., 2001; Young and Lesperance, 2001; Lindeberg, 2013a; Lindeberg, 2021) and Gabor filters by Marcelja (1980); Jones and Palmer (1987a, 1987b); Porat and Zeevi (1988). Gaussian derivatives have, in turn, been used as primitives in theoretical models of early visual processing by Lowe (2000); May and Georgeson (2007); Hesse and Georgeson (2005); Georgeson et al. (2007); Hansen and Neumann (2008); Wallis and Georgeson (2009); Wang and Spratling (2016); Pei et al. (2016); Ghodrati et al. (2017); Kristensen and Sandberg (2021); Abballe and Asari (2022); Ruslim et al. (2023) and Wendt and Faul (2024).

Hubel and Wiesel (1959, 1962, 1968, 2005) pioneered the study of simple and complex cells. The properties of simple cells have been further characterized by DeAngelis et al. (1995); DeAngelis and Anzai (2004); Ringach (2002, 2004); Conway and Livingstone (2006); Johnson et al. (2008); Walker et al. (2019) and De and Horwitz (2021). Properties of complex cells have been investigated by Movshon et al. (1978); Emerson et al. (1987); Martinez and Alonso (2001); Touryan et al. (2002, 2005); Rust et al. (2005); van Kleef et al. (2010); Goris et al. (2015); Li et al. (2015) and Almasi et al. (2020), as well as modelled computationally by Adelson and Bergen (1985); Heeger (1992); Serre and Riesenhuber (2004); Einhäuser et al. (2002); Kording et al. (2004); Merolla and Boahn (2004); Berkes and Wiskott (2005); Carandini (2006); Hansard and Horaud (2011); Franciosini et al. (2019); Lindeberg (2020); Lian et al. (2021); Oleskiw et al. (2024) and Yedjour and Yedjour (2024). In this work, we will follow a specific way of modelling simple and complex cells in terms of affine Gaussian derivatives, according to the generalized affine Gaussian derivative model for visual receptive fields.

Properties of cortical maps in the primary visual cortex have, in turn, been studied in detail by Bonhoeffer and Grinvald (1991); Blasdel (1992); Maldonado et al. (1997); Koch et al. (2016); Kremkow et al. (2016); Najafian et al. (2022); Jung et al. (2022); Fang et al. (2022) and Vita et al. (2024).

There have been previous mentions regarding receptive fields with different aspect ratios (Tinsley et al., 2003; Xu et al., 2016). It has also been reported by Wilson et al. (2016) that “neurons located near pinwheel centers ... exhibit broader orientation tuning than neurons in regions of the map where neighbouring neurons exhibit similar preferences”.

According to our knowledge, there have, however, not been any previous in-depth studies in relation to a systematic expansions of receptive field shapes over the degree of elongation, nor any previously reported connections between such expansions of receptive field shapes over the degree of elongation in relation to the property of affine covariance for the family of visual receptive fields.

## Theoretical background

In this section, we: (i) give a theoretical background regarding the notion of affine covariant visual receptive fields, which constitutes the conceptual background for the hypotheses studied in this work; (ii) describe how the orientation selectivity of receptive fields is related to the degree of elongation of the receptive fields, based on an in-depth theoretical analysis of visual receptive fields according to the generalized Gaussian derivative model; and (iii) relate the computational modelling approaches taken and the contributions presented in this work to previous work in the field.

The material in this section will then constitute the conceptual background for the novel contributions in the paper, to be presented in Section 4, such that the paper can be read in a self-contained manner, without necessarily having to first read the references (Lindeberg, 2023, 2025), which this paper closely builds upon.

### Affine covariant visual receptive fields

Let us represent the spatial image coordinates by $x = (x_1, x_2)^T \in {\mathbb {R}}^2$ and centered affine spatial transformations in the 2-D image domain as

1 $$\begin{aligned} x' = A \, x, \end{aligned}$$

where *A* represents any non-singular $2 \times 2$ matrix and $x' = (x'_1, x'_2)^T \in {\mathbb {R}}^2$ denotes the transformed image coordinates.

Then, an affine transformed image $f' :{\mathbb {R}}^2 \rightarrow {\mathbb {R}}$ of an original image $f' :{\mathbb {R}}^2 \rightarrow {\mathbb {R}}$ is defined according to

2 $$\begin{aligned} f'(x') = f(x). \end{aligned}$$

Let us denote the space of sufficiently smooth functions corresponding to continuous image data ${\mathbb {R}}^2 \rightarrow {\mathbb {R}}$ by *V*. With the affine transformation operator $\mathcal{T}_A: V \rightarrow V$ from this space onto itself, we can write the affine transformation as

3 $$\begin{aligned} f' = \mathcal{T}_A \, f. \end{aligned}$$

The property of affine covariance then means that the results of either:

- applying an affine transformation $x' = A \, x$ to an image *f* and then applying a receptive field $\mathcal{R}': V \rightarrow V$ to the affine transformed image $f'$, or
- applying a related receptive field $\mathcal{R}: V \rightarrow V$ to the original image *f* and then applying an affine transformation $\mathcal{T}_A$ to that output,

will lead to the same result, such that

4 $$\begin{aligned} \mathcal{R}' \, \mathcal{T}_A\, f = \mathcal{T}_A \, \mathcal{R} \, f, \end{aligned}$$

where the affine covariant property of the receptive field family means that for every receptive field $\mathcal{R}$ in the receptive field family, there exists a possibly transformed receptive field $\mathcal{R}'$ within the same family, specifically determined according to the actual value of the affine transformation matrix *A*, such that the above relationship is guaranteed to hold, for some transformed receptive field $\mathcal{R}'$ as a function of the original receptive field $\mathcal{R}$ and the affine transformation matrix *A*.

The property of affine covariance thus means that the family of receptive fields is well-behaved with regard to spatial affine transformations, in the sense that affine transformations commute with the operation of computing outputs from the family of receptive fields.

In Lindeberg (2021, 2023, 2025b), it is argued that such affine covariant properties constitute an essential property of spatial receptive fields, as well as for the spatial components in joint spatio-temporal receptive fields. Specifically, the receptive fields, according to the generalized Gaussian derivative model for visual receptive fields, to be used below, obey such affine covariant properties.

The property of the degree of elongation of the receptive fields, to be studied in detail in this work, spans a 1-D variability within the full 4-D variability of general affine transformations. Thus, it constitutes one of the degrees of freedom in the variability of transformed receptive field shapes of $\mathcal{R}'$, that will be generated by subjecting an original receptive field $\mathcal{R}$ to the 4-D variability of general affine transformation matrices *A*; see Lindeberg (2025a) for further details.

### The hypothesis about affine covariant receptive fields

If we assume that the visual system should implement affine covariant receptive fields (Lindeberg, 2023 Section 3.2), then the property of affine covariance would make it possible to compute better estimates of local surface orientation, compared to a visual system that does not implement affine covariance, or a sufficiently good approximation thereof.

A general motivation for the wider underlying working hypothesis about affine covariance can be stated as follows: If the population of receptive fields would support affine covariance in the primary visual cortex, or sufficiently good approximations thereof, then such an ability would support the possibility of computing affine invariant image representations at higher levels in the visual hierarchy (Lindeberg, 2013b). Alternatively, one could consider also sufficiently good approximations thereof, over restricted subspaces or subdomains of the most general forms of full variability under spatial affine transformations of the visual stimuli.

Fundamentally, we cannot expect the visual perception system to implement full affine invariance. For example, from the well-known experience, that it is much harder to read text upside-down, it is clear that the visual perception system cannot be regarded as invariant to spatial rotations in the image domain. However, from the expansion of the orientations of visual receptive fields according to the pinwheel structure of higher mammals, we can regard the population of receptive fields as supporting local rotational covariance.

When we look at a slanted surface in the world, we can get a robust and stable perception of its surface texture under substantial variations of the slant angle. This robustness of visual perception under stretchings of image patterns that correspond to non-uniform scaling transformations (the perspective effects on a slanted surface patch can, to first-order of approximation, be modelled as a stretching of the image pattern along the tilt direction in image space, complemented with a uniform scaling transformation). If the visual receptive fields would span a variability under such spatial stretching transformations, then such a variability would precisely correspond to a variability in the anisotropy, or the degree of elongation, of the receptive fields.

From these theoretical motivations, one may hence raise the question whether biological vision in higher mammals would have developed computational mechanisms in the visual pathway that could be modelled in terms of affine covariance. In (Lindeberg, 2023 Hypothesis 1 in Section 3.2.1), it has been proposed whether the visual receptive fields in the primary visual cortex may comprise an “expansion of spatial receptive field shapes over a larger part of the affine group than mere rotations or uniform scale changes”.

The main topic of this paper is to investigate the possible validity of this hypothesis with respect to the degree of freedom of spatial affine transformations corresponding to non-uniform scaling transformations over the image domain, and corresponding to receptive field shapes of different degrees of elongation.

### Connections between the orientation selectivity and the degree of elongation of the receptive fields for the generalized Gaussian derivative model for visual receptive fields

For modelling the receptive fields in the primary visual cortex, we will use the generalized Gaussian derivative model for receptive fields (Lindeberg, 2021).

#### Idealized models for simple cells

We will model the purely spatial component of the receptive fields for simple cells as (Lindeberg, 2021 Equation (23); see Figure 7 in that reference for illustrations)

5 $$\begin{aligned} \begin{array}{c} T_{\text{ simple }}(x_1, x_2;\; \sigma _{\varphi }, \varphi , \Sigma _{\varphi }, m) \\ = T_{\varphi ^m,\text{ norm }}(x_1, x_2;\; \sigma _{\varphi }, \Sigma _{\varphi }) = \sigma _{\varphi }^{m} \, \partial _{\varphi }^{m} \left( g(x_1, x_2;\; \Sigma _{\varphi }) \right) , \end{array}\end{aligned}$$

and with joint spatio-temporal receptive fields of the simple cells according to (Lindeberg, 2021 Equation (25); see Figures 10-11 in that reference for illustrations)

6 $$\begin{aligned} {\begin{matrix} T_{\text{ simple }}(x_1, x_2, t;\; \sigma _{\varphi }, \sigma _t, \varphi , v, \Sigma _{\varphi }, m, n) \end{matrix}}\nonumber \\ {\begin{matrix}&= T_{{\varphi }^m, {\bar{t}}^n,\text{ norm }}(x_1, x_2, t;\; \sigma _{\varphi }, \sigma _t, v, \Sigma _{\varphi }) \end{matrix}}\nonumber \\ {\begin{matrix}&= \sigma _{\varphi }^{m} \, \sigma _t^{n} \, \partial _{\varphi }^{m} \,\partial _{\bar{t}}^n \left( g(x_1 - v_1 t, x_2 - v_2 t;\; \Sigma _{\varphi }) \, h(t;\; \sigma _t) \right) , \end{matrix}} \end{aligned}$$

where

- $\varphi \in [-\pi , \pi ]$ is the preferred orientation of the receptive field,
- $\sigma _{\varphi } \in {\mathbb {R}}_+$ is the amount of spatial smoothing,
- $\partial _{\varphi }^m = (\cos \varphi \, \partial _{x_1} + \sin \varphi \, \partial _{x_2})^m$ is an *m*:th-order directional derivative operator, in the direction $\varphi$,
- $\Sigma _{\varphi }$ is a $2 \times 2$ symmetric positive definite covariance matrix, with one of its eigenvectors in the direction of $\varphi$,
- $g(x;\; \Sigma _{\varphi })$ is a 2-D affine Gaussian kernel with its shape determined by the covariance matrix $\Sigma _{\varphi }$
  7 $$\begin{aligned} g(x;\; \Sigma _{\varphi }) = \frac{1}{2 \pi \sqrt{\det \Sigma _{\varphi }}} e^{-x^T \Sigma _{\varphi }^{-1} x/2} \end{aligned}$$
  for $$x = (x_1, x_2)^T \in {\mathbb {R}}^2$$,
- $\sigma _t \in {\mathbb {R}}_+$ is the amount of temporal smoothing,
- $v = (v_1, v_2)^T \in {\mathbb {R}}^2$ is a local motion vector, in the direction $\varphi$ of the spatial orientation of the receptive field,
- $\partial _{\bar{t}}^n = (\partial _t + v_1 \, \partial _{x_1} + v_2 \, \partial _{x_2})^n$ is an *n*:th-order velocity-adapted temporal derivative operator, and
- $h(t;\; \sigma _t)$ is a temporal Gaussian kernel with standard deviation $\sigma _t$ over time $t \in {\mathbb {R}}$.

This model builds upon the regular Gaussian derivative model for purely spatial receptive fields proposed by Koenderink (1984); Koenderink and van Doorn (1987, 1992) and previously used for modelling biological fields by Young and his co-workers (1987; Young et al., 2001; Young and Lesperance, 2001). Here, that regular Gaussian derivative model is additionally generalized to affine covariance, according to Lindeberg (2013a, 2021).

#### Idealized models for complex cells

To model complex cells with a purely spatial dependency, we will use a quasi-quadrature measure of the form (Lindeberg, 2020 Equation (39))

8 $$\begin{aligned} \mathcal{Q}_{\varphi ,\text{ spat },\text{ norm }} L = \sqrt{L_{\varphi ,\text{ norm }}^2 + C_{\varphi } \, L_{\varphi \varphi ,\text{ norm }}^2}, \end{aligned}$$

where

- $L_{\varphi ,\text{ norm }}$ and $L_{\varphi \varphi ,\text{ norm }}$ constitute the results of convolving the input image with scale-normalized directional affine Gaussian derivative operators of orders 1 and 2:
  9 $$\begin{aligned} \begin{array}{c} L_{\varphi ,\text{ norm }}(x_1, x_2;\; \sigma _{\varphi }, \Sigma _{\varphi }) =\\ = T_{\varphi ,\text{ norm }}(x_1, x_2;\; \sigma _{\varphi }, \Sigma _{\varphi }) * f(x_1, x_2), \end{array}\end{aligned}$$
  10 $$\begin{aligned} \begin{array}{c} L_{\varphi \varphi ,\text{ norm }}(x_1, x_2;\; \sigma _{\varphi }, \Sigma _{\varphi }) =\\ = T_{\varphi \varphi ,\text{ norm }}(x_1, x_2;\; \sigma _{\varphi }, \Sigma _{\varphi }) * f(x_1, x_2), \end{array}\end{aligned}$$
- $C_{\varphi } > 0$ is a weighting factor between first and second-order information.

This model constitutes an affine Gaussian derivative analogue of the energy model of complex cells developed by Adelson and Bergen (1985) and Heeger (1992), and is consistent with the observation that receptive fields analogous to first- *vs.* second-order derivatives occur in pairs in biological vision (Valois et al., 2000), with close analogies to quadrature pairs, as defined in terms of a Hilbert transform (Bracewell 1999, pp. 267–272).

Complex cells with a joint spatio-temporal dependency will, in turn, be modelled as

11 $$\begin{aligned} \begin{array}{c} (\mathcal{Q}_{\varphi ,\text{ vel },\text{ norm }} L) = \sqrt{L_{\varphi ,\text{ norm }}^2 + \, C_{\varphi } \, L_{\varphi \varphi ,\text{ norm }}^2}, \end{array}\end{aligned}$$

where

12 $$\begin{aligned} \begin{array}{c} L_{\varphi ,\text{ norm }}(x_1, x_2, t;\; \sigma _{\varphi }, \sigma _t, v, \Sigma _{\varphi }) =\\ = T_{\varphi ,\text{ norm }}(x_1, x_2, t;\; \sigma _{\varphi }, \sigma _t, v, \Sigma _{\varphi }) * f(x_1, x_2, t), \end{array}\end{aligned}$$

13 $$\begin{aligned} \begin{array}{c} L_{\varphi \varphi ,\text{ norm }}(x_1, x_2, t;\; \sigma _{\varphi }, \sigma _t, v, \Sigma _{\varphi }) =\\ = T_{\varphi \varphi ,\text{ norm }}(x_1, x_2, t;\; \sigma _{\varphi }, \sigma _t, v, \Sigma _{\varphi }) * f(x_1, x_2, t), \end{array}\end{aligned}$$

with the underlying space-time separable spatio-temporal receptive fields $T_{\varphi ^m, t^n,\text{ norm }}(x_1, x_2, t;\; \sigma _{\varphi }, \sigma _t, v, \Sigma _{\varphi })$ according to Eq. (6) for $n = 0$.

**Fig. 3:**
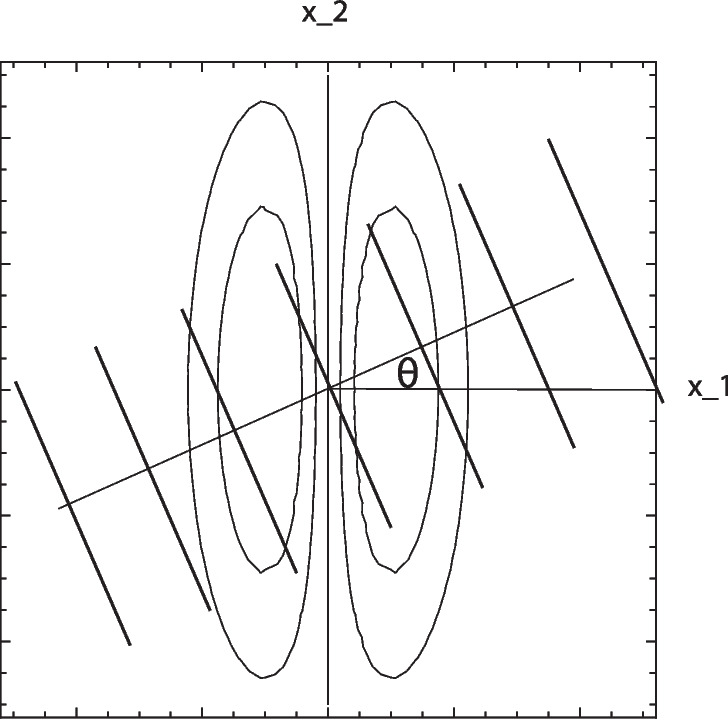
Schematic illustration of the sine wave probe used for defining the orientation selectivity curve, by using a receptive field model with the fixed preferred orientation $\varphi = 0$, and then exposing the receptive field to sine waves for different inclination angles $\theta$. (Horizontal axis: spatial coordinate $x_1$. Vertical axis: spatial coordinate $x_2$)

**Fig. 4:**
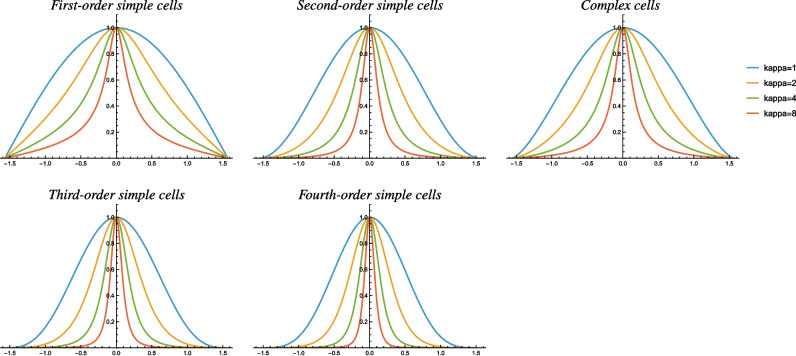
Graphs of the orientation selectivity for the idealized models of (top left) simple cells in terms of first-order directional derivatives of affine Gaussian kernels, (top middle) simple cells in terms of second-order directional derivatives of affine Gaussian kernels, (top right) complex cells in terms of directional quasi-quadrature measures that combine the first- and second-order simple cell responses in a Euclidean way for $C_{\varphi } = C_t = 1/\sqrt{2}$, (bottom left) simple cells in terms of third-order directional derivatives of affine Gaussian kernels and (bottom middle) simple cells in terms of fourth-order directional derivatives of affine Gaussian kernels, and shown for different values of the ratio $\kappa$ between the spatial scale parameters in the vertical *vs.* the horizontal directions. Observe how the degree of orientation selectivity varies strongly depending on the eccentricity $\epsilon = 1/\kappa$ of the receptive fields. (Horizontal axes: orientation $\theta \in [-\pi /2, \pi /2]$. Vertical axes: Amplitude of the receptive field response relative to the maximum response obtained for $\theta = 0$)

#### Orientation selectivity curves for the idealized receptive field models

In Lindeberg (2025), the responses of the above purely spatial models of receptive fields are calculated with respect to a static sine wave with orientation $\theta \in [-\pi , \pi ]$ and phase $\beta \in [-\pi , \pi ]$ of the form (see Fig. 3)

14 $$\begin{aligned} f(x_1, x_2) = \sin \left( \omega \cos (\theta ) \, x_1 + \omega \sin (\theta ) \, x_2+ \beta \right) . \end{aligned}$$

Additionally, the responses of the above joint spatio-temporal models of receptive fields are calculated with respect to a moving sine wave of the form

15 $$\begin{aligned} \begin{array}{c} f(x_1, x_2, t) = \\ = \sin \left( \omega \cos (\theta ) \, (x_1 - u_1 t) + \omega \sin (\theta ) \, (x_2 - u_2 t) + \beta \right) , \end{array}\end{aligned}$$

with the velocity vector $(u_1, u_2)^T$ parallel to the inclination angle $\theta$ of the grating, such that $(u_1, u_2)^T = (u \cos \theta , u \sin \theta )^T$ for $u = \sqrt{u_1^2 + u_2^2}$.

In summary, the theoretical analysis in Lindeberg (2025) shows that the resulting orientation selectivity curves for the first-order simple cells, second-order simple cells and complex cells, respectively, will be of the forms:

16 $$\begin{aligned} {\begin{matrix} r_{\text{ simple },1}(\theta )&= \frac{\left| \cos \theta \right| }{\sqrt{\cos ^2 \theta + \kappa ^2 \sin ^2\theta }}, \end{matrix}}\end{aligned}$$

17 $$\begin{aligned} {\begin{matrix} r_{\text{ simple },2}(\theta )&= \frac{\cos ^2 \theta }{\cos ^2 \theta + \kappa ^2 \sin ^2\theta }, \end{matrix}}\end{aligned}$$

18 $$\begin{aligned} {\begin{matrix} r_{\text{ complex }}(\theta )&= \frac{\left| \cos \theta \right| ^{3/2}}{\left( \cos ^2 \theta + \kappa ^2 \sin ^2\theta \right) ^{3/4}}, \end{matrix}} \end{aligned}$$

with similar angular dependencies within each class for both the purely spatial receptive fields and the joint spatio-temporal receptive fields, where

19 $$\begin{aligned} \kappa = \frac{\sigma _2}{\sigma _1} \end{aligned}$$

denotes the ratio between the scale parameters $\sigma _2 \in {\mathbb {R}}_+$ and $\sigma _1 \in {\mathbb {R}}_+$ in the vertical and horizontal directions of the affine Gaussian kernel that determines the spatial shape of the receptive field.

The top row in Fig. 4 shows graphs of these orientation selectivity curves, where we can clearly see how the orientation selectivity becomes more narrow for increasing values of the scale parameter ratio $\kappa$, thus establishing a direct link between the elongation and the degree of orientation selectivity for these idealized receptive fields.

In Appendix C in the supplementary material, we additionally extend these results to orientation selectivity for purely spatial third-order simple cells and fourth-order simple cells of the forms

20 $$\begin{aligned} {\begin{matrix} r_{\text{ simple },3}(\theta )&= \frac{\left| \cos \theta \right| ^3}{(\cos ^2 \theta + \kappa ^2 \sin ^2\theta )^{3/2}}, \end{matrix}}\end{aligned}$$

21 $$\begin{aligned} {\begin{matrix} r_{\text{ simple },4}(\theta )&= \frac{\cos ^4 \theta }{(\cos ^2 \theta + \kappa ^2 \sin ^2\theta )^2}, \end{matrix}} \end{aligned}$$

and with examples of graphs of these curves shown in the bottom row in Fig. 4 for a few values of the scale ratio parameter $\kappa$. Also for the third- and fourth-order receptive fields, the orientation selectivity curves become more narrow, both with increasing values of the scale parameter ratio $\kappa$ and with increasing order of spatial differentiation.

## Modelling the orientation selectivity properties of simple and complex cells in the primary visual cortex in terms of affine Gaussian derivative based receptive fields with a variability in eccentricity

### Interpretation of the connection between the orientation selectivity and the elongation of receptive fields in relation to biological measurements

In this section, we will compare the results of the theoretical predictions in Section 3.3 with biological results concerning the orientation selectivity of visual neurons.

#### Interpretation of the measurements about broad vs. sharp orientation selectivity of neurons by Nauhaus et al. (2008)

**Fig. 5:**
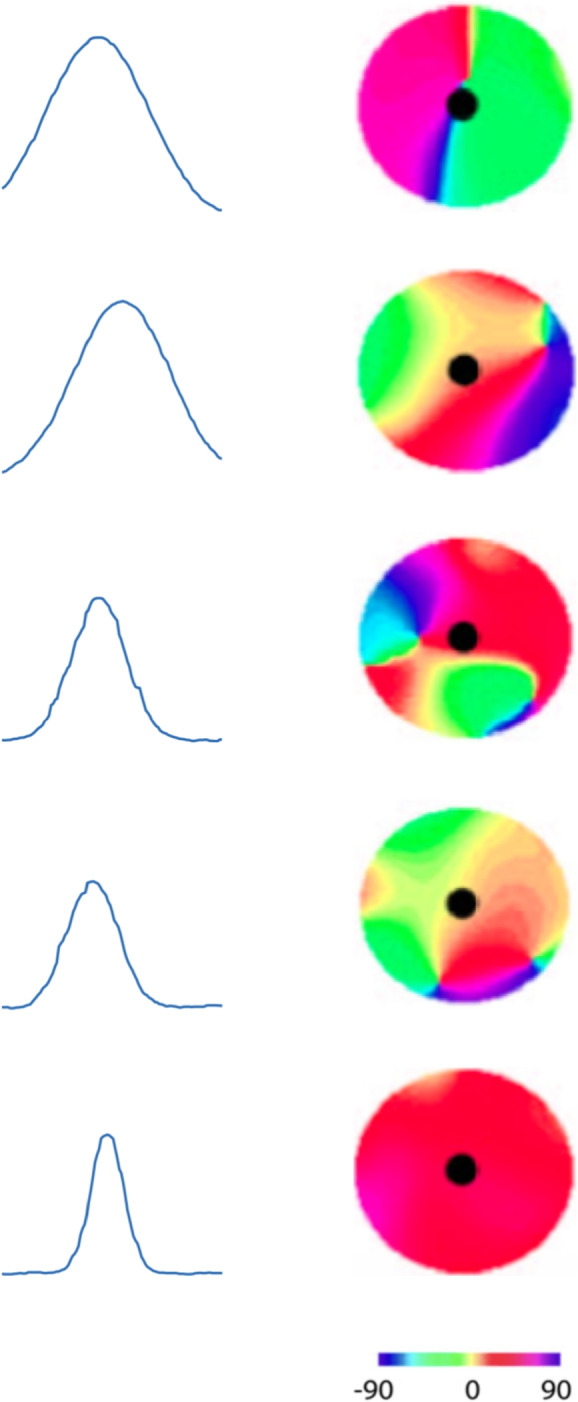
Schematic depiction of results from measurements of the orientation tuning of neurons, at different positions in the visual cortex, adapted from Nauhaus et al. (2008), showing how the orientation tuning changes from broad to sharp, and thus higher degree of orientation selectivity, with increasing distance from the pinwheels, consistent with the qualitative behaviour that would be obtained if the ratio $\kappa$, between the scale parameters in underlying affine Gaussian smoothing step in the idealized models of spatial and spatio-temporal receptive fields, would increase when moving away from the centers of the pinwheels on the cortical surface

Nauhaus et al. (2008) have measured the orientation tuning of neurons at different positions in the primary visual cortex for monkey and cat. They found that the orientation tuning is broader near the pinwheel centers and sharper in regions of homogeneous orientation preference, see specifically Figure 2 in Nauhaus et al. (2008). Figure 5 shows a schematic depiction of their results, where the degree of orientation selectivity changes^3^ from broad to sharp with increasing distance from the pinwheel center (from top to bottom in the figure), however, more clearly visible in the original Figure 2 in Nauhaus et al. (2008). This behaviour is also in agreement with the observation by Wilson et al. (2016) that “neurons located near pinwheel centers ... exhibit broader orientation tuning than neurons in regions of the map where neighbouring neurons exhibit similar preferences”.

Thus, first of all, we can see that the variability in the orientation selectivity of visual neurons is consistent with a variability in the degree of elongation of the receptive fields, as predicted by the underlying normative theory of visual receptive fields, and also consistent with the more general hypotheses, that the family of receptive fields ought to be expanded over the degrees of freedom of affine image transformations to be affine covariant.

Secondly, this qualitative behaviour is consistent with what would be the result if the ratio $\kappa$ between the two scale parameters of the underlying affine Gaussian kernels would increase from a lower to a higher value, when moving away from the centers of the pinwheels on the cortical surface. Thus, by combination with the experimental results by Nauhaus et al. (2008) and Wilson et al. (2016), the presented theory leads to a prediction about a systematic variability in the eccentricity or the elongation of the receptive fields in the primary visual cortex, which for the case of pinwheel structures, would be consistent with a variability in the eccentricity or the elongation of the receptive fields from the centers of the pinwheels towards the periphery.

A highly interesting quantitative measurement to perform, in view of these theoretical results, would hence be to fit parameterized models of the orientation selectivity, according to Eqs. (16), (17), (18), (20) and (21)

22 $$\begin{aligned} r_{\lambda }(\theta ) = \left( \frac{\left| \cos \theta \right| }{\sqrt{\cos ^2 \theta + \kappa ^2 \sin ^2\theta }} \right) ^{\lambda } \end{aligned}$$

for $\lambda \in {\mathbb {R}}$ to orientation tuning curves of the form recorded by Nauhaus et al. (2008), to get estimates of the distribution of the parameter $\kappa$ over a sufficiently large population of visual neurons, under the assumption that the spatial components of the biological receptive fields can be well modelled by affine Gaussian derivatives.^4^

If we would assume that it would be unlikely for the receptive fields to have as strong variability in their orientational selectivity properties as a function of the positions of the neurons in relation to the pinwheel structure, as reported in this study, without also having a strong variability in their eccentricity. Then, by combining the theoretical analysis in this article with the biological results by Nauhaus et al. (2008), that would serve as possible indirect support for the hypothesis concerning an expansion of receptive field shapes over variations in the ratio between the two scale parameters of spatially anisotropic receptive fields.

**Fig. 6:**
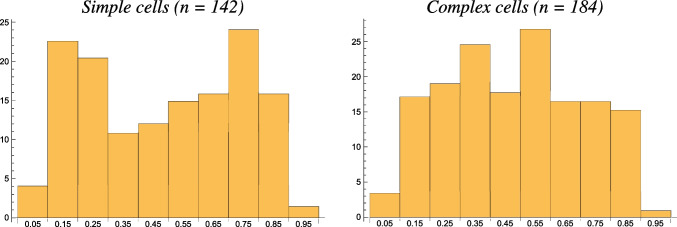
Schematic depictions of the shapes of the distributions of the absolute value of the resultant $|R| \in [0, 1]$ according to Eq. (23) for the directional selectivity of visual neurons over populations of simple cells and complex cells, respectively, in the primary visual cortex, adapted from neurophysiological recordings of Macaque monkeys by Goris et al. (2015). (Horizontal axes: 10 quantized bins over the resultant $|R| \in [0, 1]$. Vertical axes: approximate bin counts)

#### Qualitative interpretation of the measured orientation selectivity histograms by Goris et al. (2015)

These predictions are furthermore consistent with existing biological results by Goris et al. (2015), concerning the distribution of the degree of orientation selectivity of the neurons in the primary visual cortex. By measuring the absolute value $|R| \in {\mathbb {R}}$ of the complex-valued resultant, given by

23 $$\begin{aligned} R = \frac{\int _{\theta = - \pi }^{\pi } r(\theta ) \, e^{2 i \theta } d\theta }{\int _{\theta = - \pi }^{\pi } r(\theta ) \, d\theta }, \end{aligned}$$

for each visual neuron, and then computing a normalized histogram of these measurements (see Fig. 6 for a schematic depiction of the results in Fig. 1B in Goris et al. (2015)) demonstrate a substantial variability in the orientation selectivity of the receptive fields of simple cells and complex cells in the primary visual cortex.

**Fig. 7:**
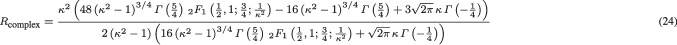
Closed-form expression for the resultant *R* according to Eq. (23) calculated for the orientation selectivity curves Eq. (18) for our idealized models of complex cells, valid for the purely spatial model in Eq. (8) and the joint spatio-temporal model Eq. (11). The function $_2F_1(a, b; c; z)$ denotes the hypergeometric function ${\text {Hypergeometric2F1}}[a, b, c, z]$ in Mathematica, while $\Gamma (z)$ represents Euler’s Gamma function

This consistency can be demonstrated by computing the closed-form expression for the absolute value of the resultant of the orientation selectivity curves according to Eqs. (16)–(18) and Eqs. (20)–(21):

- for the first-order idealized models of simple cells
  25 $$\begin{aligned} {\begin{matrix} R_{\text{ simple },1}&= \frac{\int _{\theta = -\pi /2}^{\pi /2} \frac{ \cos \theta }{\sqrt{\cos ^2 \theta + \kappa ^2 \sin ^2\theta }} \, \cos 2 \theta \, d\theta }{\int _{\theta = -\pi /2}^{\pi /2} \frac{ \cos \theta }{\sqrt{\cos ^2 \theta + \kappa ^2 \sin ^2\theta }} \, d\theta } \end{matrix}}\nonumber \\ {\begin{matrix}&= \frac{\kappa \left( \kappa \cosh ^{-1} \kappa -\sqrt{\kappa ^2-1}\right) }{\left( \kappa ^2-1\right) \cosh ^{-1} \kappa }, \end{matrix}} \end{aligned}$$
- for the second-order idealized models of simple cells
  26 $$\begin{aligned} {\begin{matrix} R_{\text{ simple },2}&= \frac{\int _{\theta = -\pi /2}^{\pi /2} \frac{\cos ^2 \theta }{\cos ^2 \theta + \kappa ^2 \sin ^2\theta } \, \cos 2 \theta \, d\theta }{\int _{\theta = -\pi /2}^{\pi /2} \frac{\cos ^2 \theta }{\cos ^2 \theta + \kappa ^2 \sin ^2\theta } \, d\theta } \end{matrix}}\nonumber \\ {\begin{matrix}&= \frac{\kappa }{\kappa +1}, \end{matrix}} \end{aligned}$$
- for the idealized models of complex cells
  27 $$\begin{aligned} {\begin{matrix} R_{\text{ complex }}&= \frac{\int _{\theta = -\pi /2}^{\pi /2} \frac{\cos ^{3/2} \theta }{\left( \cos ^2 \theta + \kappa ^2 \sin ^2\theta \right) ^{3/4}} \, \cos 2 \theta \, d\theta }{\int _{\theta = -\pi /2}^{\pi /2} \frac{\cos ^{3/2} \theta }{\left( \cos ^2 \theta + \kappa ^2 \sin ^2\theta \right) ^{3/4}} \, d\theta }, \end{matrix}} \end{aligned}$$
  with the explicit expression for that result in Fig. 7,
- for the third-order idealized models of simple cells
  28 $$\begin{aligned} {\begin{matrix} R_{\text{ simple },3}&= \frac{\int _{\theta = -\pi /2}^{\pi /2} \frac{ \cos ^3 \theta }{(\cos ^2 \theta + \kappa ^2 \sin ^2\theta )^{3/2}} \, \cos 2 \theta \, d\theta }{\int _{\theta = -\pi /2}^{\pi /2} \frac{ \cos ^3 \theta }{(\cos ^2 \theta + \kappa ^2 \sin ^2\theta )^{3/2}} \, d\theta } \end{matrix}}\nonumber \\ {\begin{matrix}&= \frac{\kappa \left( \sqrt{\kappa ^2-1} \left( \kappa ^2+2\right) -3 \kappa \cosh ^{-1}(\kappa )\right) }{\left( \kappa ^2-1\right) \left( \kappa \sqrt{\kappa ^2-1}-\cosh ^{-1}(\kappa )\right) }, \end{matrix}} \end{aligned}$$
- and for the fourth-order idealized models of simple cells
  29 $$\begin{aligned} {\begin{matrix} R_{\text{ simple },4}&= \frac{\int _{\theta = -\pi /2}^{\pi /2} \frac{\cos ^4 \theta }{(\cos ^2 \theta + \kappa ^2 \sin ^2\theta )^2} \, \cos 2 \theta \, d\theta }{\int _{\theta = -\pi /2}^{\pi /2} \frac{\cos ^4 \theta }{(\cos ^2 \theta + \kappa ^2 \sin ^2\theta )^2} \, d\theta } \end{matrix}}\nonumber \\ {\begin{matrix}&= \frac{\kappa \, (\kappa +3)}{(\kappa +1) (\kappa +2)}. \end{matrix}} \end{aligned}$$

**Fig. 8:**
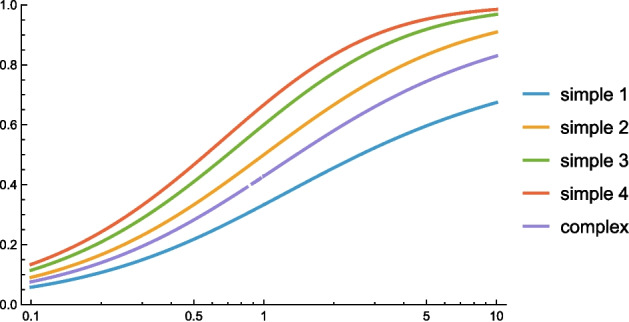
Graphs of the resultant *R* for idealized models of (i) a first-order simple cell according to Eq. (25), (ii) a second-order simple cell according to Eq. (26), (iii) a third-order simple cell according to Eq. (28), (iv) a fourth-order simple cell according to Eq. (29) and (v) a complex cell according to Eq. (27) on a log-linear scale, with the horizontal axis parameterized in terms of the logarithm $K = \log \kappa$ of the scale parameter ratio $\kappa$ in the affine Gaussian derivative model of visual receptive fields. (Horizontal axes: scale parameter ratio $\kappa$. Vertical axes: resultant *R*)

Let us additionally reparameterize these curves in terms of a logarithmic parameterization $K = \log \kappa$ of the scale parameter ratio $\kappa$, which leads to graphs shown in Fig. 8. Then, we can see that the experimentally obtained distributions in Fig. 6 appear to be reasonably consistent^5^ with the assumption of a rather uniform distribution over the logarithmically parameterized scale parameter ratio $K = \log \kappa$.

Such a parameterization would specifically constitute a canonical parameterization, if one would simplify^6^ the 2-D joint distribution of receptive field shapes over the scale parameter ratio $\kappa$ and the orientation $\varphi$ into two independent 1-D distributions over the scale parameter ratio $\kappa$ and the orientation $\varphi$, respectively, in the idealized model of visual receptive fields according to the generalized Gaussian derivative framework.

Thus, also these biological results are qualitatively consistent with the working hypothesis about an expansion over the degree of elongation of the receptive fields in the primary visual cortex, as would be implied from the assumption of a family of affine covariant visual receptive fields.

#### Quantitative modelling of the measured orientation selectivity histograms by Goris et al. (2015)

To aim at more detailed *quantitative* modelling of the experimentally recorded histograms of the resultant measure of the orientation selectivity curves, as reported by Goris et al. (2015) and as schematically reproduced in Fig. 6, we need to consider that there are more free parameters in the modelling stage to determine, based on the following arguments:

- One basic question concerns what range of values of the scale parameter ratio $\kappa$ would be spanned by the receptive fields in the primary visual cortex.^7^
- Another basic question concerns the distribution of receptive fields with respect to the order of spatial differentiation. To reproduce an idealized model of a histogram of the resultant *R* for a population of simple cells, as shown in the top part of Fig. 6, we would therefore have to a assume a distribution of receptive fields over different orders of spatial differentiation.

**Fig. 9:**
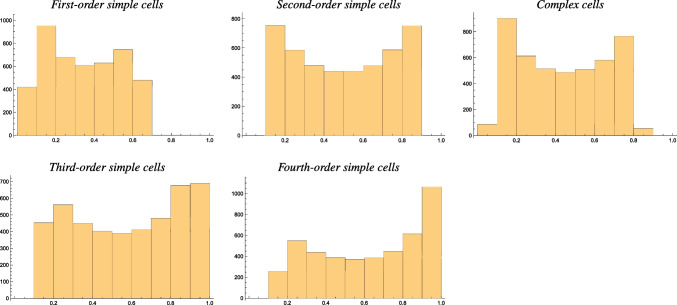
Examples of histograms of the resultant *R* over populations of (top left) first-order simple cells, (top middle) second-order simple cells, (top right) complex cells, (bottom left) third-order simple cells and (bottom right) fourth-order simple cells, accumulated over a uniform logarithmic distribution of the scale parameter ratio $\kappa$ over the interval $\kappa \in [1/\kappa _{\max }, \kappa _{\max }]$ for $\kappa _{\max } = 8$. (Horizontal axes: bin over the resultant $R \in [0, 1]$. Vertical axes: number of receptive fields in this bin in a discrete simulation)

To illustrate to what extent the distributions of the resultant will be influenced by receptive fields for different orders of spatial differentiation, Fig. 9 shows histograms of the resultant accumulated for idealized models of simple cells of orders 1, 2, 3 and 4 as well as for the idealized model of complex cells. For generating these graphs, we have created a uniform distribution of the scale parameter ratio $\kappa$ over a logarithmic scale, over the interval $\kappa \in [1/\kappa _{\max }, \kappa _{\max }]$ for the arbitrary choice of $\kappa _{\max } = 8$.

As can be seen from these graphs, the histogram over the first-order simple cells is delimited by a maximum value around $R_{\max ,1} \approx 0.7$, while the distributions for third-order and fourth-order simple cells are heavier for larger values of the resultant approaching $R \rightarrow 1$. The model used for computing histograms of the resultant for the idealized models of complex cells does, however, not very well reproduce the shape of the biologically obtained histogram, thus indicating that the model for the complex cells may be overly simplified^8^ for the purpose of reproducing the shape of the resultant histogram. See the discussion in Section 6 in Lindeberg (2025) for a number of suggested ways to extend that model.

**Fig. 10:**
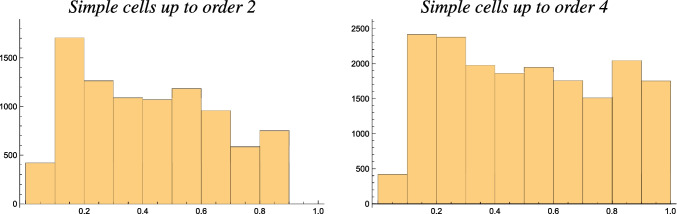
Examples of combined histograms of the resultant *R* over populations of simple cells of different order (left) up to order 2 and (right) up to order 4, accumulated over a uniform logarithmic distribution of the scale parameter ratio $\kappa$ over the interval $\kappa \in [1/\kappa _{\max }, \kappa _{\max }]$ for $\kappa _{\max } = 8$. (Horizontal axes: bin over the resultant $R \in [0, 1]$. Vertical axes: number of receptive fields in this bin in a discrete simulation)

Figure 10 shows additional results of combining the resultant for populations of simple cells over different orders of spatial differentiation, either up to order 2 or up to order 4, here assuming the same number of neurons for all the different orders of spatial differentiation.

With ample reservation from the fact that this theoretical analysis is conceptually simplified in a number of ways,^9^ as can be seen from these results, the combined histograms give rise to a bump in the histograms for lower values of the resultant *R*, in qualitative agreement with the biological results by Goris et al. (2015) and as reproduced in Fig. 6. Furthermore, to obtain something that would look like a small bump for larger values of the resultant *R*, the modelling situation with receptive fields up to order 4 gives a closer similarity to the biological results by Goris et al. (2015) compared to the model based on receptive fields up to order 2.

Assuming that the modelling simplifications do not significantly affect the qualitative nature of the results, from the presented analysis it thus appears as if:

- The histograms of the resultant of the simple cells in the biological experiments (Goris et al., 2015) could be rather well explained by the receptive fields in the primary visual cortex of Macaque monkeys having a variability over the degree of elongation.
- The non-uniform nature of the experimentally obtained histogram of the resultant *R* for the simple cells could be explained better by assuming that receptive fields should be present up to a spatial differentiation order up to 4 than up to a spatial differentiation order of 2.

#### Summary of the interpretations and modelling results of the biological experiments by Nauhaus et al. (2008) and by Goris et al. (2015)

To conclude, the biological results by Nauhaus et al. (2008); Goris et al. (2015) and Wilson et al. (2016) are clearly consistent with an expansion over the degree of elongation of the receptive field shapes in the primary visual cortex.

Based on these results we propose that, beyond an expansion over rotations, as is performed in current models of the pinwheel structure of visual receptive fields (Bonhoeffer and Grinvald, 1991; Blasdel, 1992; Swindale, 1996; Petitot, 2003; Koch et al., 2016; Kremkow et al., 2016; Baspinar et al., 2018; Najafian et al., 2022; Liu and Robinson, 2022), also an explicit expansion over the eccentricity $\epsilon$ of the receptive fields (the inverse of the parameter $\kappa$) should be included, when modelling the pinwheel structure in the visual cortex.

Possible ways, by which an explicit dependency on the eccentricity of the receptive fields could be incorporated into the modelling of pinwheel structures, will be outlined in more detail in the following treatment regarding more specific biological hypotheses.

### Explicit testable hypotheses for biological experiments

Based on the above theoretical analysis, with its associated theoretical predictions, we propose that it would be highly interesting to perform experimental characterization and analysis based on joint estimation of

- orientational selectivity,
- receptive field eccentricity,
- orientational homogeneity and
- location of the neuron in the visual cortex in relation to the pinwheel structure,

in the primary visual cortex of animals with clear pinwheel structures, to determine if there is a variability in the eccentricity or elongation of the receptive fields, and specifically if the degree of elongation increases with the distance from the centres of the pinwheels towards periphery, as arising as one possible interpretation of combining the theoretical results about orientation selectivity of affine Gaussian receptive fields in this article with the biological results by Nauhaus et al. (2008).

**Fig. 11:**
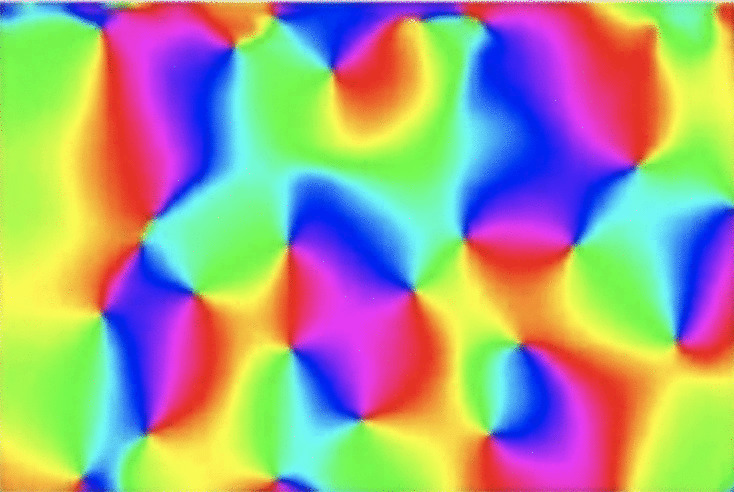
Orientation map in the primary visual cortex of cat, as recorded by Koch et al. (2016) (OpenAccess), with the orientation preference of the receptive fields encoded in terms of colours, and demonstrating that the visual cortex performs an explicit expansion of the receptive field shapes over spatial image orientations

**Fig. 12:**
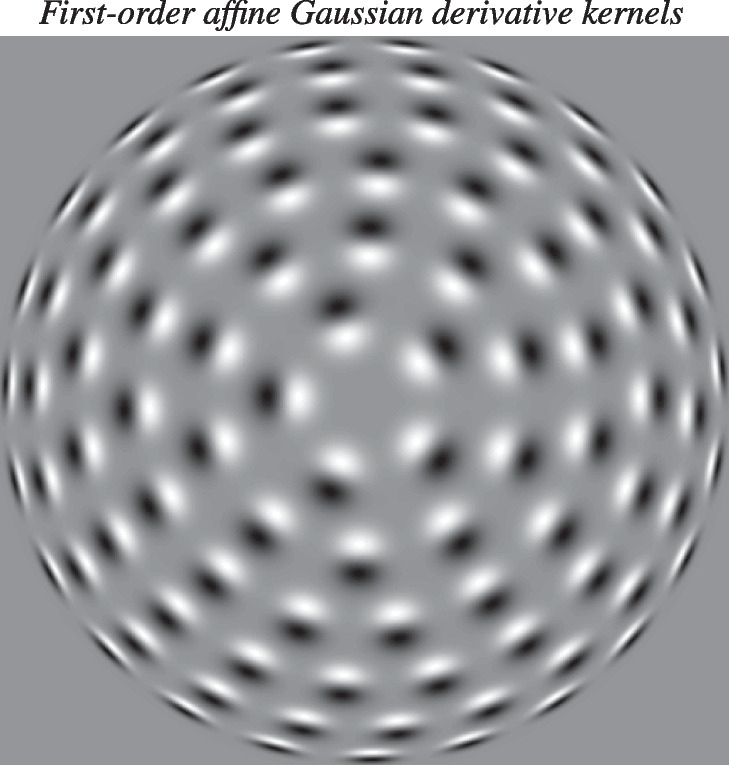
Distribution of first-order affine Gaussian derivative kernels of the form Eq. (5) for different spatial covariance matrices $\Sigma$, with their elements parameterized according to $C_{11} = \sigma _1^2 \, \cos ^2 \varphi + \sigma _2^2 \, \sin ^2 \varphi$, $C_{12} = C_{21} = (\sigma _1^2 - \sigma _2^2) \cos \varphi \, \sin \varphi$, and $C_{22} = \sigma _1^2 \, \sin ^2 \varphi + \sigma _2^2 \, \cos ^2 \varphi$, with the larger spatial scale parameter $\sigma _2$ in this illustration held constant, while the smaller scale parameter $\sigma _1$ varies as $\sigma _1 = \sigma _2/\kappa$, according to a distribution on a hemisphere. The spatial directional derivatives are, in turn, defined according to $\partial _{\varphi } = \cos \varphi \, \partial _{x_1} + \sin \varphi \, \partial _{x_2}$. The possible additional variability of the scale parameters, beyond their ratio $\kappa$, is, however, not explicitly addressed in this paper. From a biological viewpoint, one could, indeed, possibly think that it might be easier to keep the smaller scale parameter $\sigma _1$ constant, and let the larger scale parameter $\sigma _2$ increase towards the periphery, since a higher degree of orientation selectivity can then be achieved by just integrating over successively larger support regions in the image space. The important aspect of this illustration is rather that the eccentricity $\kappa$ increases from the most isotropic image position towards the periphery. With regard to the possible connection to the pinwheel structure in the primary visual cortex, the center in this figure would correspond to the center of the pinwheel, whereas the periphery would correspond to the boundaries of the part of the visual cortex that is closest to the center of one particular pinwheel. (Reprinted from Lindeberg (2023) (OpenAccess)

If additionally, reconstructions of the receptive field shapes could be performed for the receptive fields probed during such a systematic investigation of the difference in response characteristics with the distance from the pinwheel centers, and if the receptive fields could additionally be reasonably well modelled according to the generalized Gaussian model for receptive fields studied and used in this paper. Then, it would be interesting to investigate if the shapes of the affine Gaussian components of these receptive fields would span a larger part of the affine group, than the span over mere image orientations, as already established in the orientation maps of the visual cortex, as characterized by Bonhoeffer and Grinvald (1991); Blasdel (1992) and others, see Fig. 11 for an illustration. A working hypothesis in the paper concerns to investigate whether the primary visual cortex could *additionally* perform an expansion over the eccentricity or the elongation of the spatial components of the receptive fields.

If we would lay out the shapes of affine Gaussian receptive fields according to the shapes of their underlying spatial covariance matrices $\Sigma$, then we would for a fixed value of their size (the spatial scale parameter) obtain a distribution of the form shown in Fig. 12. That directional distribution is, however, in a certain aspect redundant, since opposite orientations on the unit circle are represented by two explicit copies, where the corresponding receptive fields are either equal, for receptive fields corresponding to spatial directional derivatives of even order, or of opposite sign for derivatives of even order. Could it be established that the receptive fields shapes, if expanded over a variability over eccentricity or elongation, for animals that have a clear pinwheel structure, have a spatial distribution that can somehow be related to such an idealized distribution, if we collapse opposite image orientations to the same image orientation, by *e.g.* a double-angle mapping $\varphi \mapsto 2 \varphi$?

Notably the variability of the spatial covariance matrices in the affine Gaussian derivative model comprises a variability over two spatial scale parameters $\sigma _1$ and $\sigma _2$, while the theoretical analysis of the orientation selectivity properties studied in this article has mainly concerned their ratio $\kappa = \sigma _2/\sigma _1$. Hence, the illustration in Fig. 12 should not be taken as a literal prediction, even if reduced by a double-angle representation. In Fig. 12, the larger scale parameter $\sigma _1$ is held constant, for convenience of graphical illustration, as obtained by mapping the uniformly sized receptive fields from a uniform distribution on the hemisphere. More generally, one could also conceive other distributions as possible, such as instead keeping the smaller eigenvalue $\sigma _2$ constant from the center towards the periphery.

To conclude, we propose to state the following testable hypotheses for biological experiments:

#### Hypothesis 1

**(Variability in eccentricity)** Let $\sigma _{\varphi }$ and $\sigma _{\bot \varphi }$ be the characteristic lengths in the preferred directions of an orientation selective simple cell in the primary visual cortex. Then, over a population of such simple cells, there is a substantial variability in their eccentricity ratio $\epsilon = \sigma _{\varphi }/\sigma _{\bot \varphi }$.

#### Hypothesis 2

**(Variability in eccentricity coupled to orientational homogeneity)** Assuming that Hypothesis 1 holds, let $\epsilon$ denote the eccentricity of a simple cell in the primary visual cortex, and let *H* be a measure of the homogeneity in the orientation preference of its surrounding neurons. Then, over a population of simple cells, there is a systematic connection between $\epsilon$ and *H*.

#### Hypothesis 3

**(Variability in eccentricity coupled to the pinwheel structure)** Assuming that Hypothesis 1 holds, let $\epsilon$ denote the eccentricity of a simple cell in the primary visual cortex. Then, over a population of simple cells, there is a systematic connection between $\epsilon$ and the distance from the nearest pinwheel center.

If Hypothesis 3 would hold, then we could also sharpen this hypothesis further as:

#### Hypothesis 4

**(Increase in elongation with increasing distance from the centers of the pinwheels)** Assuming that Hypothesis 3 holds, let $\epsilon$ denote the eccentricity measure of a simple cell in the primary visual cortex defined such that $\epsilon = 1$ if the characteristic lengths of the spatial receptive fields are equal, and tending towards zero as the characteristic lengths differ more and more. Then, over a population of simple cells, the eccentricity measure decreases from the center of the pinwheel towards the periphery.

Note that the latter explicit hypotheses have been expressed on a general form, of not explicitly assuming that the biological receptive fields can be well modelled according to the generalized Gaussian derivative model for receptive fields. The essential factor in the definitions is only that it should be possible to define estimates of the characteristic lengths $\sigma _{\varphi }$ and $\sigma _{\bot \varphi }$, so as to be able to define a measure of the eccentricity $\epsilon$.

If either Hypothesis 2 or Hypotesis 3 would hold, then we could also explicitly state the following hypothesis:

#### Hypothesis 5

**(Pinwheel structure more structured than a mere expansion over spatial orientations)** The pinwheel structure comprises an, at least, two-dimensional variability of receptive field shapes, beyond an expansion over spatial orientations, also an expansion over the eccentricity of the receptive fields in the primary visual cortex.

For simplicity, we have above expressed these hypotheses for the case of simple cells, for which it is easiest to define the measures $\sigma _{\varphi }$ and $\sigma _{\bot \varphi }$ of the characteristic lengths, because of the linearity of the receptive fields. Provided that corresponding measures of characteristic lengths could also be in a sufficiently well-established way be defined also for non-linear complex cells, corresponding explicit hypotheses could also be formulated for complex cells.

It should finally be stressed that, in this treatment, we have not considered the binocular aspects of the pinwheel structure. In Hypothesis 5, the variability of the pinwheel structure over contributions from the left and the right eyes should therefore not be counted as a property to contribute to the terminology “more structured”.

### Quantitative measurements for detailed characterization

To further characterize possible relationships between the orientational selectivity, receptive field eccentricity, orientational homogeneity, and the location of the neuron in relation to the pinwheel structure in the primary visual cortex, we would also propose to characterize the possible relationships between these entities in terms of:

#### Quantitative measurement 1: (Relationship between orientational selectivity and receptive field eccentricity)

Graph or scatter diagram showing how a quantitative measure of orientational selectivity is related to a quantitative measure of receptive field eccentricity, accumulated over a sufficiently large population of neurons.

#### Quantitative measurement 2: (Relationship between orientational homogeneity and receptive field eccentricity)

Graph or scatter diagram showing how a quantitative measure of orientational homogeneity is related to a quantitative measure receptive field eccentricity, accumulated over a sufficiently large population of neurons.

#### Quantitative measurement 3: (Relationship between receptive field eccentricity and the pinwheel structure)

Graph or scatter diagram showing how a quantitative measure of receptive field eccentricity depends on the distance to the nearest pinwheel center, accumulated over a sufficiently large population of neurons.

#### Quantitative measurement 4: (Relationship between receptive field eccentricity and the pinwheel structure)

Two-dimensional map showing how a quantitative measure of receptive field eccentricity relates to a two-dimensional map of the orientation preference over the same region in the primary visual cortex, with the center of the pinwheel structure explicitly marked, again accumulated over a sufficiently large population of neurons.

If the above theoretically motivated biological hypotheses could be investigated experimentally, and if the above quantitative measurements of receptive field characteristics could be performed. Then, it could be judged if the prediction from the presented theoretical analysis about a systematic variability in receptive field eccentricity, with a possible relationship to the pinwheel structure, could be either experimentally supported or rejected. In a corresponding manner, such a judgement could also answer if the receptive fields in the primary visual cortex could be regarded as spanning a larger part of the affine group, than an expansion over mere rotations in the image domain.

## Relations to other types of models of visual receptive fields

Concerning the analysis presented in the previous section, it should be emphasized that it constitutes a theoretical modelling step at a *functional* mathematical level. The complementary theoretical explanation in terms of a partial affine covariance property regarding the degree of freedom corresponding to non-uniform scaling transformation does furthermore operate at a coarse level of abstraction, regarding theoretically desirable properties of an idealized vision system.

Hence, this theoretical explanation should not be regarded as in conflict with other possible theoretical explanations in terms of learning of spatial receptive fields by sparse coding, which may also lead to learned receptive fields with different degrees of elongation (Olshausen and Field, 1996, 1997; Rehn and Sommer, 2007; Zylberberg et al., 2011; King et al., 2013). Similarly, our conceptual explanation is neither in conflict with a more fine-grained computational explanation of varying receptive field shapes in terms of different “On” or “Off” responses within the receptive fields, as proposed by Martinez et al. (2005) and neurophysiologically investigated by Kremkow et al. (2016) and Jansen et al. (2019).

Instead, our proposed explanation is that the notion of affine covariance or more generally covariance to geometric image transformations may be an essential factor in the development of biological vision systems. As natural images are formed in the retinas of visual observers, the statistical properties of the image data that reach the visual sensor will be strongly influenced by the structure of the natural image transformations, when 3-D objects are projected to 2-D image data from different viewing positions, viewing directions and relative motions.

The result of learning receptive fields in a biological vision agent, should thereby be strongly influenced by the structure of the natural image transformations. For a biological creature, who depends strongly on the visual perception for its survival, it does therefore seem plausible that there could be an evolutionary pressure for the biological organism to develop its vision system to be well adapted to the influence of the natural image transformations that arise when observing a dynamic world. Beyond the treatment of whether biological vision has actually developed a variability in the eccentricity of the receptive fields, our theory offers a theoretical explanation of this property, solidly grounded in a principled formal theory of visual receptive fields.

Thus, one could expect that when specific learning approaches, such as the sparse coding mechanisms considered by Olshausen and Field (1996, 1997); Rehn and Sommer (2007); Zylberberg et al. (2011) and King et al. (2013), are exposed to natural image data that contain the variabilities in image structures generated by geometric image transformations, it would be natural for such learning approaches to lead to learned receptive fields with different eccentricities. Specifically, one could then also conceive that different spatial shapes of such receptive fields to be biologically implemented in terms of different contributions form “On” and “Off” subregions, as considered by Martinez et al. (2005); Kremkow et al. (2016) and Jansen et al. (2019).

In this respect, the presented theory in this paper thus offers a theoretical explanation of these phenomena at a more general and functional level of abstraction, by assuming an idealized vision system that adapts its processing to the inherent structures of natural image structures as influenced by natural image transformations. The property that the receptive fields in the primary visual cortex should have a variability in their eccentricity is then a direct consequence of this general assumption, *via* the assumption of affine covariance. This could then also conceptually explain other possible explanations in terms of more explicit learning mechanisms or specific neurophysiological mechanisms.

In Appendices A and B in the supplementary material we additionally: (i) give relations to a corresponding analysis based on Gabor models of the visual receptive fields, and (ii) describe relations to more detailed and fine-grained models of the primary visual cortex.

## Summary and discussion

We have compared results from theoretical analysis of the orientation selectivity properties of the affine Gaussian derivative model (Section 3.3) with experimental results by Nauhaus et al. (2008) on broadly vs. sharply tuned visual neurons (Fig. 5) and by Goris et al. (2015) on rather uniform distributions of the resultant values from orientation selectivity curves (Fig. 6). Thereby, we have found potential support for one of the dimensions of variability in a biological hypothesis formulated in Lindeberg (2023), stating that the family of receptive field shapes ought to span the degrees of freedom in the natural geometric image transformations. This potential support rests on the assumption, that it should be unlikely for the population of receptive fields to show strong variability in orientation selectivity, without also showing similar variability in eccentricity or elongation.

Without explicitly relying on expressing such an explicit assumption, regarding whether the visual receptive fields in the primary visual cortex could be well modelled by affine Gaussian derivative based receptive fields, we can, however, firmly state that the biological measurements performed by Nauhaus et al. (2008) and by Goris et al. (2015) are, in combination with the theoretical results summarized in Section 3.3, consistent with the hypothesis that the receptive fields should span a variability in the eccentricity of the receptive fields, and more widely consistent with the hypothesis about affine covariant receptive fields.

Furthermore, from the results presented in Section 4.1.3, that the orientation selectivity histograms accumulated by Goris et al. (2015) appear to be better explained by a population of receptive fields up to order 4 than by receptive fields up to order 2, one may speculate if it would be appropriate to put additional focus of neurophysiological experiments on exploring the presence of visual receptive fields of higher order, compared to the receptive fields of lower order more commonly reported in the neurophysiological literature.

If we apply a similar type of assumption-based logical reasoning to the pinwheel structure in the primary visual cortex, then such a reasoning, based on the results by Nauhaus et al. (2008) and by Wilson et al. (2016), that the orientation selectivity appears to vary strongly from the centers of the pinwheels towards the periphery. Then, this implies that the pinwheel structure in the visual cortex would, beyond an explicit expansion over image orientations, also comprise an explicit expansion over the the degree of elongation of the receptive fields. Based on these predictions, we propose to consider explicit dependencies on a variability in the eccentricity of the receptive fields, when modelling the pinwheel structure in the primary visual cortex.

Strictly, and formally, the results from such logical inference could, however, only be regarded as theoretical predictions, to generate explicit hypothesis concerning the distribution of receptive field characteristics in these respects. To raise the question of determining if these theoretical predictions would firmly hold in reality, we propose that the testable explicit biological hypotheses formulated in Section 4.2 could be used to, in neurophysiological experiments, either verify or reject the overall hypothesis, concerning possible variabilities in the eccentricity of the receptive fields in the primary visual cortex of higher mammals. These predictions could also be used to explore hypotheses about possible connections between such variabilities in the eccentricity or the elongation and other receptive field characteristics, in particular in relation to the pinwheel structure in the primary visual cortex of higher mammals.

Notably, the overall analysis in the paper is carried based on a framework that reflects functional properties of an idealized vision system, in terms of covariance under geometric image transformations, and using mathematical analysis as the main tool. Thereby, the analysis in the paper is performed without any extensive numerical simulations of explicit neuron models, which would otherwise have to contain many parameters, some of which commonly are unobservable and would have to be estimated indirectly from data.

Concerning possible limitations in the hypothetical reasoning stages used for possible logical inference and for formulating the explicit biological hypotheses above, the logical reasoning based on connections between the eccentricity and the degree of elongation of the receptive fields depend on explicitly stated assumptions regarding whether the biological receptive fields could be reasonably well modelled by affine Gaussian derivative based receptive fields. To be able to draw possible further conclusions, the possible validity of those hypothetical logical reasoning stages could, however, break down, if there would be other external factors, not covered by the theoretical model, that could also strongly influence the orientation selectivity of the receptive fields. The possible applicability of the hypothetical logical reasoning stages above thus, ultimately, depends on the possible agreement between the model and biological data, and can only be taken further by performing complementary neurophysiological experiments, to ultimately judge if the theoretically based predictions, stated in Section 4.2, would be applicable to actual biological neurons.

Furthermore, while the treatment in this paper has been concerned with species that have orientation maps and a pinwheel structure, a very interesting follow-up question would then also concern if corresponding results would extend to species that do not have structured orientation maps or a pinwheel structure, and then specifically which such species. For example, Niell and Stryker (2008) have shown that the mouse visual cortex has developed mechanisms of orientation selectivity, although not having pinwheel structures. For analyzing that topic in detail, further results from biological experiments and on other species would, however, be necessary, why we leave that topic to future work.

## Supplementary Information

Below is the link to the electronic supplementary material.

Supplementary file 1 (pdf 149 KB)

## Data Availability

No datasets were generated or analysed during the current study.

## Footnotes

1 For an in-depth theoretical analysis of the degrees of freedom of 2-D spatial affine transformations in relation to the degrees of freedom of the shapes of 2-D visual receptive fields in terms of affine Gaussian derivatives, see (Lindeberg, 2025a).

2 The terminology used throughout this paper of “spanning a variability”, is similar to the terminology in mathematics concerning a set of vectors that together span a vector space or a subspace. More generally, from the viewpoint of the wider classes of geometric image transformations in the generalized Gaussian derivative theory for visual receptive fields, one could consider these geometric image transformations to have different degrees of freedom. These degrees of freedom in the geometric image transformations will then correspond to different degrees of freedom in the shapes of the visual receptive fields, as induced by covariance properties of the receptive fields. A set of such degrees of freedom taken together will thereby span a corresponding variability in the shapes of the visual receptive fields.

3 In Fig. 5, the orientation selectivity is widest in the top row, corresponding to the center of the pinwheel. The orientation selectivity is most narrow in the bottom row, corresponding to a position in the visual cortex far away from the center of the pinwheel. Then, for the three intermediate rows, the orientation selectivity is intermediate, and corresponding to intermediate positions in the visual cortex some distance away from the centers of the pinwheels.

4 At the point of writing this article, the author does, however, not have access to the explicit raw data that would be needed to perform such an analysis.

5 If the logarithmic parameterization of the scale parameter ratio $\kappa$ would be a perfect prior for resultant measure *R*, then these graphs would be pure linear functions, so that the corresponding histograms over the resultant *R* would then be constant.

6 More generally, one could instead conceive a uniform joint distribution on a hemisphere, as conceived in Figure 8 in Lindeberg (2021), which regarding first-order spatial derivatives then leads to a distribution of spatial receptive field shapes of the form shown in Fig. 12, and possibly complemented with additional priors to account for how important different local surface orientations in the environment would be for the perceptual process, as well as how densely the space of combined image orientations $\varphi$ and scale parameter ratios $\kappa$ would need to be sampled, to support sufficiently good approximations of covariance over that submanifold for the local image measurements performed by the family of spatial receptive fields. In this treatment, we do, however, simplify this problem, by instead considering a uniform distribution over a logarithmic transformation of the scale parameter ratio $\kappa$, which is also easier to handle in closed form calculations, and which may be regarded as a coarse approximation, to compensate for gross phenomena with regard to a non-uniform distribution of receptive field shapes over the scale parameter ratio $\kappa$.

7 In the graphs shown in Fig. 8, we have used a range of the scale parameter ratio $\kappa$ over a factor 10 from the unit value 1, which results in a maximum value of *R* for the idealized first-order model of a simple cell of about 0.67. In contrast, for the idealized second-order model of a simple cell, *R* reaches a maximum value of *R* about 0.91, while for the complex cell, *R* assumes a maximum value of about 0.83. For the third-order simple cell the maximum value of *R* is about 0.97 and for the fourth-order simple cell the maximum value of *R* is about 0.98. If this range of scale parameter ratios would be reduced to a lower span, then the range of the possible values of *R* would be reduced, while the range would be expanded if a wider range of scale ratios $\kappa$ would be implemented.

8 In this context, it should be remarked that there is strong conceptual difference between the idealized models for simple cells *vs.* the idealized models of complex cells. The idealized model for simple cells has been determined in a theoretically principled manner from axiomatic derivations, and also been matched to biological measurements of simple cells, whereas the idealized model for complex cells has been chosen as an as straightforward way as possible for combining the responses of odd-shaped and even-shaped simple cells.

9 The reservation that we have to state concerning this theoretical modelling of the resultant histograms accumulated by Goris et al. (2015), however, is that the analysis is simplified in the following 5 major ways: (i) In the current state, we do not have any principled biological arguments for choosing particular values of the parameters $\kappa _{\min }$ and $\kappa _{\max }$ that determine the range of the scale parameter ratio $\kappa$, where different choices of these parameters may affect the shapes of the combined histograms of the resultant *R*. (ii) The choice of a logarithmic distribution over the scale parameter $\kappa$ does, as previously mentioned, neglect any co-dependency with respect to the distribution over the orientation angle $\varphi$. (iii) The experimental conditions used when accumulating the orientation selectivity curves for biological neurons may not be identical to the conditions under which the theoretically derived orientation selectivity curves have been derived. (iv) The assumption about equal numbers of receptive fields for the different orders of spatial differentiation may not necessarily hold in reality. (v) Our computations of the resultant *R* for the receptive fields are based on an idealized noise free model, while there additionally could be sources of noise in the biological experiments as well as modelling errors between the receptive fields of the actual biological neurons and our idealized receptive fields.
