# Supplementary material for: Do the receptive fields in the primary visual cortex span a variability over the degree of elongation of the receptive fields?
Source: J Comput Neurosci. 2025 Jun 20;53(3):397–417. doi: 10.1007/s10827-025-00907-4 (PMC12417286; doi:10.1007/s10827-025-00907-4)
Supplement: Supplementary file 1 — (pdf 149 KB) [file 10827_2025_907_MOESM1_ESM.pdf]

Submitted to Journal of Computational Neuroscience

Supplementary material to "Do the receptive fields in the primary visual cortex span a variability over the degree of elongation of the receptive fields?"

Tony Lindeberg

Abstract This document contains supplementary material for the paper

> Lindeberg (2025) "Do the receptive fields in the primary visual cortex span a variability over the degree of elongation of the receptive fields?"

published in Journal of Computational Neuroscience:

- Appendix A gives relations to a corresponding analysis based on Gabor models of the visual receptive fields.
- Appendix B describes relations to more detailed and fine-grained models of the primary visual cortex.
- Appendix C derives orientation selectivity properties for third- and fourth-order simple cells.

A Relations to a corresponding analysis based on Gabor models

In view of the presented analysis of a predicted expansion of receptive field shapes over the degree of elongation of the receptive fields, one may naturally ask if a corresponding type of analysis could also be carried out for the Gabor model of visual receptive fields. For such a purpose, let us consider an affine Gabor model of the form

$$T_{\text{even}}(x_1, x_2;\ \sigma_1, \sigma_2, \nu) = \frac{1}{2\pi\sigma_1\sigma_2}\, e^{-x_1^2/2\sigma_1^2 - x_2^2/2\sigma_2^2} \cos(\nu\, x_1), \tag{30}$$

$$T_{\text{odd}}(x_1, x_2;\ \sigma_1, \sigma_2, \nu) = \frac{1}{2\pi\sigma_1\sigma_2}\, e^{-x_1^2/2\sigma_1^2 - x_2^2/2\sigma_2^2} \sin(\nu\, x_1), \tag{31}$$

where σ_1 and σ_2 are the spatial scale parameters and ν the angular frequency. For simplicity, we have here oriented the affine Gabor model to the image orientation $\varphi = 0$.

In Lindeberg (2025) Section 5, it is shown that if we couple the spatial scale parameters σ_1 and σ_2 in that model according to $\sigma_2 = \kappa\,\sigma_1$ for $\kappa > 1$, then larger values of the scale parameter κ will lead to more narrow orientation selectivity properties. There is, however, also another degree of freedom in the affine Gabor model, implying that variations in the angular frequency ν may also strongly affect the orientation selectivity properties of the receptive fields.

Specifically, comparing the explicit expressions for the orientation selectivity curves derived for the affine Gabor model in Lindeberg (2025)

$$A_{\text{even}}(\theta) = \frac{1}{2}\left(e^{2\nu^2\sigma_1^2\cos\theta} + 1\right) e^{-\nu^2\sigma_1^2\cos^2\left(\frac{\theta}{2}\right)(\kappa^2-1)(1-\cos\theta)}, \tag{32}$$

$$A_{\text{odd}}(\theta) = \frac{1}{2}\left(e^{2\nu^2\sigma_1^2\cos\theta} - 1\right) e^{-\nu^2\sigma_1^2\cos^2\left(\frac{\theta}{2}\right)(\kappa^2-1)(1-\cos\theta)}. \tag{33}$$

to the explicit expressions for the orientation selectivity curves for the generalized Gaussian derivative model for visual receptive fields, summarized to the form according to Equation (22) in the main body of the paper

$$r_\lambda(\theta) = \left(\frac{|\cos\theta|}{\sqrt{\cos^2\theta + \kappa^2\sin^2\theta}}\right)^\lambda \tag{34}$$

for a few discrete values of λ, we see that the relationship between the degree of elongation of a receptive field and the orientation selectivity properties of the receptive field is rather complex for the affine Gabor model, while it is very direct for the generalized Gaussian derivative model for visual receptive fields.

Thus, if we assume that the population of the visual neurons would be described by an affine Gabor model, and if

The support from the Swedish Research Council (contract 2022-02969) is gratefully acknowledged.

Computational Brain Science Lab, Division of Computational Science and Technology, KTH Royal Institute of Technology, SE-100 44 Stockholm, Sweden. E-mail: tony@kth.se. ORCID: 0000-0002-9081-2170.

we observe variations in the orientation selectivity properties of the neurons, in terms of "more narrow" or "less narrow", then it is, in fact, *not possible* to logically infer that those variations in the orientation selectivity would necessarily have to be due to variations in the scale parameter ratio κ, since those variations in the orientation selectivity properties could also be due to variations in the other degree of freedom, determined by the interactions between the angular frequency ν, the spatial scale parameter σ_1 and the scale parameter ratio κ.

Regarding the use of a Gabor model for the visual receptive fields, it should also be stressed that the Gabor model is a purely spatial model, while the biological receptive fields in the primary visual cortex do also have strong temporal dependencies. A further advantage of the generalized Gaussian derivative model for visual receptive fields in that context, is that it also comprises theoretically principled models for joint spatio-temporal receptive fields.

Additionally, we have in Lindeberg (2025) shown that the orientation selectivity curves for the joint spatio-temporal receptive field models up to order 2 according to Equations (6) and (11) in the main body of the paper lead to similar orientation selectivity curves according to Equations (16)–(18) in the main body of the paper as for the corresponding purely spatial models according to Equations (5) and (8) in the main body of the paper. In this respect, the analysis based on the generalized Gaussian derivative model for visual receptive fields has a much wider domain of validity, compared to a corresponding analysis based on the affine Gabor model for visual receptive fields, since its results hold also for the more realistic domain of joint spatio-temporal receptive fields.

B Relations to more detailed and fine-grained models of the primary visual cortex

Compared to more more detailed and fine-grained quantitative models of the primary visual cortex (Troyer *et al.* 1998, Zhu *et al.* 2009, Shu *et al.* 2015, Schmidt *et al.* 2018, Einevoll *et al.* 2019, Billeh *et al.* 2020, Chariker *et al.* 2022, Antolík *et al.* 2024), a major conceptual difference of the presented highly idealized and mathematically principled model, is that the predictions and the results can be obtained with just straightforward theoretical modelling and analysis, and essentially, thus, be obtained in closed form, as opposed to the results from numerical simulations of comparably much more complex models, then implying a need for both (i) further assumptions, explicit or implicit, for formulating the actual models, as well as for (ii) setting the values of the possibly rather large number of parameters in such more complex models. Specifically, it is most often the case that some parameters are not experimentally measured and therefore need to be obtained by parameter estimation (parameter tuning) according to functional criteria, thereby necessitating these functional criteria to be formulated, further complicating the use of complex models.

Whereas one could possibly argue that the particle simulation approach, used for accumulating the receptive field histograms in Sections 4.1.2 and 4.1.3 in the main body of the paper, from explicitly generated samples from the distributions over the scale parameter ratio κ, could also be regarded as constituting numerical simulations with a certain numerical uncertainty, due to sampling issues of the underlying distributions, it would, in principle, also be possible to instead obtain more accurate estimates of the corresponding receptive field histograms without using any use of such sampled distributions. Instead, one can numerically solve the equations that determine the boundaries of the quantization bins in the distributions of the receptive fields, which, in turn, form the histograms as integrals of the underlying distributions over the resulting bin regions in the parameter space. Such an approach would then lead to results without any dependence on sampling issues regarding the model. In the experimental results reported in this paper, such possible sampling issues would nevertheless be expected to be very small, due to the rather large number of samples underlying the accumulation of the sampled histograms. Furthermore, the focus of this paper is on qualitative properties with regard to the distributions of receptive fields over different degrees of elongation.

While large scale models could be very valuable, if they could be properly aligned with biological measurements, more detailed and fine-grained models of the primary visual cortex, do on the other hand require numerical calculations to be evaluated, and do therefore rely on explicit numerical simulations for getting the results. Hence, a large number of larger-scale simulations would be needed to get corresponding results as presented in this paper. Then, also those results would furthermore depend on the assumptions used for constructing those models as well as on the choices of the complementary parameters in those substantially more complex models, that they are based on. To interpret such corresponding results, regarding the possible validity of the hypothesis of an expansion of receptive field shapes over the degree of elongation, rather exhaustive searches in the resulting parameter spaces may therefore be needed, to determine the regions in the rather high-dimensional parameter spaces, where the stated predictions would hold, and then also delimit the boundaries in the parameter space of such valid regions. A particular critical aspect of such an analysis, aimed at exploring whether the receptive fields would span a variability over the degree of elongation or not, would, however, concern if the architecture for the fine-grained model would, based on either implicit or explicit assumptions, either support or violate affine covariance.

If we consider the perspective of judging whether the receptive field shapes could be regarded as spanning a substantial variability over the degree of elongation. Then, the proposed model constitutes an as much simplified and idealized theoretical model as possible, while still respecting the covariance properties of the receptive fields under geometric image transformations. The model is, thus, also essentially parameter free, beyond the bounds on the scale parameter ratio κ, that were handled by symmetric intervals of the form $[1/\kappa_{\max}, \kappa_{max}]$ for the here, for purposes of illustration only, arbitrary choice of $\kappa_{\max} = 8$, and the above, for simplicity, in the absence of further information, assumed equal numbers of receptive fields corresponding to the different orders of spatial differentiation, in the composed histograms accumulated over receptive fields corresponding to multiple orders of spatial differentiation. Additionally, the underlying model for the visual receptive fields, which the presented work is based on, is theoretically very principled, by being derived in a purely axiomatic manner, and then after the axiomatic mathematical derivation *a posteriori* compared to actual neurophysiological recordings of biological receptive fields.

The model, that the presented analysis is based on, is also a purely functional model for feedforward computations in V1, and thereby decoupled from the more complex workings of the neuronal architecture in V1. From this perspective, the analysis shows that the variability in the degree of elongation of the receptive fields ought to constitute a very basic property of the receptive fields in V1, that to a first order of approximation would not necessarily explicitly depend upon such more complex complementary mechanisms.

A very interesting topic for future studies could nevertheless be to complement the presented theory with the influence of more complex neural mechanisms, such intra-V1-coupling mechanisms, the top-down influence in the visual cortex and inhibitory projections. Another interesting approach would be to integrate mechanisms for affine covariance into more fine-grained V1 models. Such more fine-grained modelling would then have the possibility to model detailed substructures in the reported orientation selectivity properties of the visual neurons, that are not comprised within the realm of our maximally idealized model. For example, Liu *et al.* (2015, 2017) have reported that the orientation tuning between the pinwheel and domain neurons in primary visual cortex may additionally depend also on the stimulus contrast and the stimulus size.[1] From a theoretical viewpoint, such complementary dependencies on stimulus contrast could be explained by the also non-linear dependencies of the receptive fields on the input stimuli, which may then lead to qualitatively different types of receptive fields from different stimuli, for example, based on mechanisms such as inhibitory tuning (Li *et al.* 2012) or, more generally, that the contrast of the visual stimuli may modulate the functional connectivity in the visual cortex (Nauhaus *et al.* 2009), see, however, also Alitto and Usrey (2004) and Nowak and Barone (2009) for complementary support regarding contrast invariant properties of the orientation tuning of the visual neurons.

[1] Furthermore, an additional dependency on stimulus size could also result from an expansion of receptive field shapes over the size of the receptive fields, as also predicted from the property of affine covariance, that underlies the normative theory of visual receptive fields, used as the theoretical foundation for this study.

In the view of such more detailed and fine-grained modelling of the neural architecture in V1, it would, indeed, be highly interesting to complement the receptive field models in our normative theory with such complementary mechanisms, to adapt it to the more fine-grained properties in the primary visual cortex. Since the exploration of such mechanisms would, however, require explicitly carrying out a substantial number of numerical simulations of much larger and more complex V1 models, we leave such extensions to future work, with the focus of this paper on purely theoretically based ways of reasoning based on our maximally simplified idealized model.

A main advantage of the presented work, based on the underlying normative theory for visual receptive fields, is therefore, that it, on the other hand, shows that it is possible to perform substantial analysis, reasoning and predictions based on a purely theoretical framework, which then enables much more compact and efficient analysis, compared to more extensive numerical simulations of more complex, detailed and fine-grained models of the primary visual cortex. As has been demonstrated, the results from this theoretical way of reasoning are also in very good qualitative agreement with the results from previously reported biological experiments.

C Derivations of orientation selectivity properties for third-order and fourth-order simple cells

For purposes in the main article, we here extend the results concerning the orientation selectivity curves for idealized models of cells according to the generalized Gaussian derivative model for visual receptive fields in (Lindeberg 2025) from first-order and second-order simple cells to also comprise third-order and fourth-order simple cells.

For simplicity, we will here restrict ourselves to purely static models of simple cells.

C.1 Third-order simple cell

Following the methodology in (Lindeberg 2025) underlying the results summarized in Section 3.3.3 in the main body of

the paper, we will express an idealized model of a simple cell with four lobes along the preferred orientation of the simple cell as a third-order scale-normalized derivative of an affine Gaussian kernel (according to Equation (5) in the main body of the paper for $m = 3$), and for convenience of the calculations choose the preferred orientation as the horizontal x_1-direction (for $\varphi = 0$) with spatial scale parameter σ_1 in the horizontal x_1-direction and spatial scale parameter σ_2 in the vertical x_2-direction, and thus with a spatial covariance matrix of the form $\Sigma_0 = \text{diag}(\sigma_1^2, \sigma_2^2)$:

$$T_{000,\text{norm}}(x_1, x_2;\ \sigma_1, \sigma_2) = \\ = \frac{\sigma_1^3}{2\pi\sigma_1\sigma_2}\,\partial_{x_1x_1x_1}\left(e^{-x_1^2/2\sigma_1^2 - x_2^2/2\sigma_2^2}\right) \\ = \frac{(3\sigma_1^2 x - x^3)}{2\pi\sigma_1^4\sigma_2}\,e^{-x_1^2/2\sigma_1^2 - x_2^2/2\sigma_2^2}. \quad (35)$$

The corresponding receptive field response can then be expressed as, after solving the convolution integral in Mathematica,

$$L_{000,\text{norm}}(x_1, x_2;\ \sigma_1, \sigma_2) = \\ = \int_{\xi_1=-\infty}^{\infty}\int_{\xi_2=-\infty}^{\infty} T_{000,\text{norm}}(\xi_1, \xi_2;\ \sigma_1, \sigma_2) \\ \times f(x_1 - \xi_1, x_2 - \xi_2)\,d\xi_1\xi_2 \\ = -\omega^3\,\sigma_1^3\cos^3(\theta)\,e^{-\frac{1}{2}\omega^2(\sigma_1^2\cos^2\theta + \sigma_2^2\sin^2\theta)} \\ \times \cos(\omega\cos(\theta)\,x_1 + \omega\sin(\theta)\,x_2 + \beta), \quad (36)$$

i.e., it corresponds to cosine wave with amplitude

$$A_{\varphi\varphi\varphi}(\theta, \omega;\ \sigma_1, \sigma_2) = \\ = \omega^3\,\sigma_1^3\cos^3(\theta)\,e^{-\frac{1}{2}\omega^2(\sigma_1^2\cos^2\theta + \sigma_2^2\sin^2\theta)}. \quad (37)$$

If we, for this modelling situation, assume that the spatial receptive field is fixed, then it follows that the amplitude of the response will strongly depend on the angular frequency ω of the sine wave. Specifically, the magnitude of the response will first increase with the angular frequency of the input stimulus, because of the factor ω. Then, it will decrease with scale because of the strong exponential decrease with ω^2.

Let us consider that a biological experiment to measure the orientation selectivity properties of a visual neuron is performed in such a way that the angular frequency of the input stimulus is varied for each inclination angle θ, and that then the result for each value orientation θ of the stimulus is only reported for the angular frequency $\hat{\omega}$ that leads to the maximum response over all the image orientations. Then, we can determine this value of $\hat{\omega}$ by differentiating $A_{\varphi}(\theta, \omega;\ \sigma_1, \sigma_2)$ with respect to ω and setting the derivative to zero, which gives:

$$\hat{\omega}_{\varphi\varphi\varphi} = \frac{\sqrt{3}}{\sqrt{\sigma_1^2\cos^2\theta + \sigma_2^2\sin^2\theta}}. \quad (38)$$

If we then insert this value into $A_{\varphi\varphi\varphi}(\theta, \omega;\ \sigma_1, \sigma_2)$, and introduce a scale parameter ratio κ such that

$$\sigma_2 = \kappa\,\sigma_1, \quad (39)$$

which gives

$$\hat{\omega}_{\varphi\varphi\varphi} = \frac{\sqrt{3}}{\sigma_1\sqrt{\cos^2\theta + \kappa^2\sin^2\theta}}. \quad (40)$$

then this gives rise to an orientation selectivity curve of the form

$$A_{\varphi\varphi\varphi,\max}(\theta,\ \kappa) = \frac{3\sqrt{3}\,\left|\cos^3\theta\right|}{e^{3/2}\left(\cos^2\theta + \kappa^2\sin^2\theta\right)^{3/2}}. \quad (41)$$

Notably, this amplitude measure is independent of the spatial scale parameter σ_1 of the receptive field. This property is a direct implication of the scale-invariant properties of differential expressions in terms of scale-normalized derivatives when using the specific value $\gamma = 1$ for the scale normalization parameter.

C.2 Fourth-order simple cell

Let us next consider an idealized model of a simple cell with five lobes along the main orientation of the receptive field, which we model with as a fourth-order scale-normalized derivative of an affine Gaussian kernel (according to Equation (5) in the main body of the paper for $m = 4$), with its preferred orientation again for convenience chosen as the horizontal x_1-direction (for $\varphi = 0$), and with a spatial scale parameter σ_1 in the horizontal x_1-direction and a spatial scale parameter σ_2 in the vertical x_2-direction, and thus again with a spatial covariance matrix of the form $\Sigma_0 = \text{diag}(\sigma_1^2, \sigma_2^2)$:

$$T_{0000,\text{norm}}(x_1, x_2;\ \sigma_1, \sigma_2) = \\ = \frac{\sigma_1^4}{2\pi\sigma_1\sigma_2}\,\partial_{x_1x_1x_1x_1}\left(e^{-x_1^2/2\sigma_1^2 - x_2^2/2\sigma_2^2}\right) \\ = \frac{(3\sigma_1^4 - 6\sigma_1^2x^2 + x^4)}{2\pi\sigma_1^5\sigma_2}\,e^{-x_1^2/2\sigma_1^2 - x_2^2/2\sigma_2^2}. \quad (42)$$

After solving the convolution integral in Mathematica, the corresponding receptive field response is then of the form

$$L_{0000,\text{norm}}(x_1, x_2;\ \sigma_1, \sigma_2) = \\ = \int_{\xi_1=-\infty}^{\infty}\int_{\xi_2=-\infty}^{\infty} T_{0000,\text{norm}}(\xi_1, \xi_2;\ \sigma_1, \sigma_2) \\ \times f(x_1 - \xi_1, x_2 - \xi_2)\,d\xi_1\xi_2 \\ = \omega^4\,\sigma_1^4\cos^4(\theta)\,e^{-\frac{1}{2}\omega^2(\sigma_1^2\cos^2\theta + \sigma_2^2\sin^2\theta)} \\ \times \sin(\omega\cos(\theta)\,x_1 + \omega\sin(\theta)\,x_2 + \beta), \quad (43)$$

i.e., a sine wave with amplitude

$$A_{\varphi\varphi\varphi\varphi}(\theta, \omega;\ \sigma_1, \sigma_2) = \\ = \omega^4\, \sigma_1^4 \cos^4(\theta)\, e^{-\frac{1}{2}\omega^2(\sigma_1^2 \cos^2\theta + \sigma_2^2 \sin^2\theta)}. \quad (44)$$

As for the previous idealized receptive field model, this expression also first increases and then increases with the angular frequency ω. Again selecting the value of $\hat{\omega}$ at which the amplitude assumes its maximum over ω gives

$$\hat{\omega}_{\varphi\varphi\varphi\varphi} = \frac{2}{\sigma_1\sqrt{\cos^2\theta + \kappa^2 \sin^2\theta}}, \quad (45)$$

which implies that the maximum amplitude over spatial scales as a function of the inclination angle θ and the scale parameter ratio κ can be written

$$A_{\varphi\varphi\varphi\varphi,\max}(\theta;\ \kappa) = \frac{16\cos^4\theta}{e^2\left(\cos^2\theta + \kappa^2 \sin^2\theta\right)^2}. \quad (46)$$

C.3 Resulting orientation selectivity curves

If we additionally normalize the orientation selectivity curves (41) and (46) to have their maximum value equal to one for the preferred orientation $\theta = 0$, we then obtain normalized orientation selectivity curves of the forms

$$r_{\text{simple},3}(\theta) = \frac{|\cos\theta|^3}{(\cos^2\theta + \kappa^2 \sin^2\theta)^{3/2}}, \quad (47)$$

$$r_{\text{simple},4}(\theta) = \frac{\cos^4\theta}{(\cos^2\theta + \kappa^2 \sin^2\theta)^2}, \quad (48)$$

and with examples of graphs of these curves, for a few values of the scale ratio parameter κ, shown in the bottow row of Figure 4 in the main body of the paper.

References

H. J. Alitto and W. M. Usrey. Influence of contrast on orientation and temporal frequency tuning in ferret primary visual cortex. *Journal of Neurophysiology*, 91(6):2797–2808, 2004.

J. Antolík, R. Cagnol, T. Rózsa, C. Monier, Y. Frégnac, and A. P. Davison. A comprehensive data-driven model of cat primary visual cortex. *PLOS Computational Biology*, 20(8):e1012342, 2024.

Y. N. Billeh, B. Cai, S. L. Gratiy, K. Dai, R. Iyer, N. W. Gouwens, R. Abbasi-Asl, X. Jia, J. H. Siegle, S. R. Olsen, C. Koch, S. Mihalas, and A. Arkhipov. Systematic integration of structural and functional data into multi-scale models of mouse primary visual cortex. *Neuron*, 106(3):388–403, 2020.

L. Chariker, R. Shapley, M. Hawken, and L.-S. Young. A computational model of direction selectivity in Macaque V1 cortex based on dynamic differences between ON and OFF pathways. *Journal of Neuroscience*, 42(16):3365–3380, 2022.

G. T. Einevoll, A. Destexhe, M. Diesmann, S. Grün, V. Jirsa, M. de Kamps, M. Migliore, T. V. Ness, H. E. Plesser, and F. Schürmann. The scientific case for brain simulations. *Neuron*, 102(4):735–744, 2019.

Y.-T. Li, W.-P. Ma, L.-Y. Li, L. A. Ibrahim, S.-Z. Wang, and H. W. Tao. Broadening of inhibitory tuning underlies contrast-dependent sharpening of orientation selectivity in mouse visual cortex. *Journal of Neuroscience*, 32(46):16466–16477, 2012.

T. Lindeberg. Orientation selectivity properties for the affine Gaussian derivative and the affine Gabor models for visual receptive fields. *Journal of Computational Neuroscience*, 53(1):61–98, 2025.

Y.-J. Liu, M. Hashemi-Nezhad, and D. C. Lyon. Contrast invariance of orientation tuning in cat primary visual cortex neurons depends on stimulus size. *The Journal of Physiology*, 593(19):4485–4498, 2015.

Y.-J. Liu, M. Hashemi-Nezhad, and D. C. Lyon. Differences in orientation tuning between pinwheel and domain neurons in primary visual cortex depend on contrast and size. *Neurophotonics*, 4(3): 031209–031209, 2017.

I. Nauhaus, L. Busse, M. Carandini, and D. L. Ringach. Stimulus contrast modulates functional connectivity in visual cortex. *Nature Neuroscience*, 12(1):70–76, 2009.

L. G. Nowak and P. Barone. Contrast adaptation contributes to contrast-invariance of orientation tuning of primate V1 cells. *PLoS ONE*, 4(3):e4781, 2009.

M. Schmidt, R. Bakker, C. C. Hilgetag, M. Diesmann, and S. J. van Albada. Multi-scale account of the network structure of Macaque visual cortex. *Brain Structure and Function*, 223(3):1409–1435, 2018.

N. Shu, Z. Gao, X. Chen, and H. Liu. Computational model of primary visual cortex combining visual attention for action recognition. *PloS ONE*, 10(7):e0130569, 2015.

T. W. Troyer, A. E. Krukowski, N. J. Priebe, and K. D. Miller. Contrast-invariant orientation tuning in cat visual cortex: Thalamocortical input tuning and correlation-based intracortical connectivity. *Journal of Neuroscience*, 18(15):908–5927, 1998.

W. Zhu, M. Shelley, and R. Shapley. A neuronal network model of primary visual cortex explains spatial frequency selectivity. *Journal of Computational Neuroscience*, 26:271–287, 2009.
